# Supplementary material for: A label-free light-scattering method to resolve assembly and disassembly of DNA nanostructures
Source: Biophys J. 2022 Oct 29;121(24):4800–9. doi: 10.1016/j.bpj.2022.10.036 (PMC9811603; doi:10.1016/j.bpj.2022.10.036)
Supplement: Document S1. Figures S1–S10 and Notes S1–S5 [file mmc1.pdf]

Biophysical Journal, Volume *121*

Supplemental information

A label-free light-scattering method to resolve assembly and disassembly of DNA nanostructures

Heini Ijäs, Tim Liedl, Veikko Linko, and Gregor Posnjak

Supplementary Information for
A label-free light scattering method to resolve assembly and disassembly of DNA nanostructures

Heini Ijäs[1,2], **Tim Liedl**[2], **Veikko Linko**[1,3,*] **& Gregor Posnjak**[2,*]

[1]Biohybrid Materials, Department of Bioproducts and Biosystems, Aalto University, P.O. Box 16100, 00076 Aalto, Finland
[2]Faculty of Physics and Center for NanoScience (CeNS), Ludwig-Maximilians-University, Geschwister-Scholl-Platz 1, 80539 Munich, Germany
[3]LIBER Center of Excellence, Aalto University, P.O. Box 15100, 00076 Aalto, Finland

Contents

Page

Supplementary notes **3**

Note S1: Spectrum of detected light (Supplementary Figure S1) . . . 3

Note S2: Scattering intensities at different wavelengths during folding (Supplementary Figure S2) . . . 4

Note S3: Background signals from buffer, staples and scaffold (Supplementary Figure S3) . . . 5

Note S4: qPCR vs. light scattering (Supplementary Figures S4-S9) . . . 6

Note S5: Non-normalized scattering intensities during DNase I digestion (Supplementary Figure S10) . . . 12

Supplementary notes

Note S1: Spectrum of detected light.

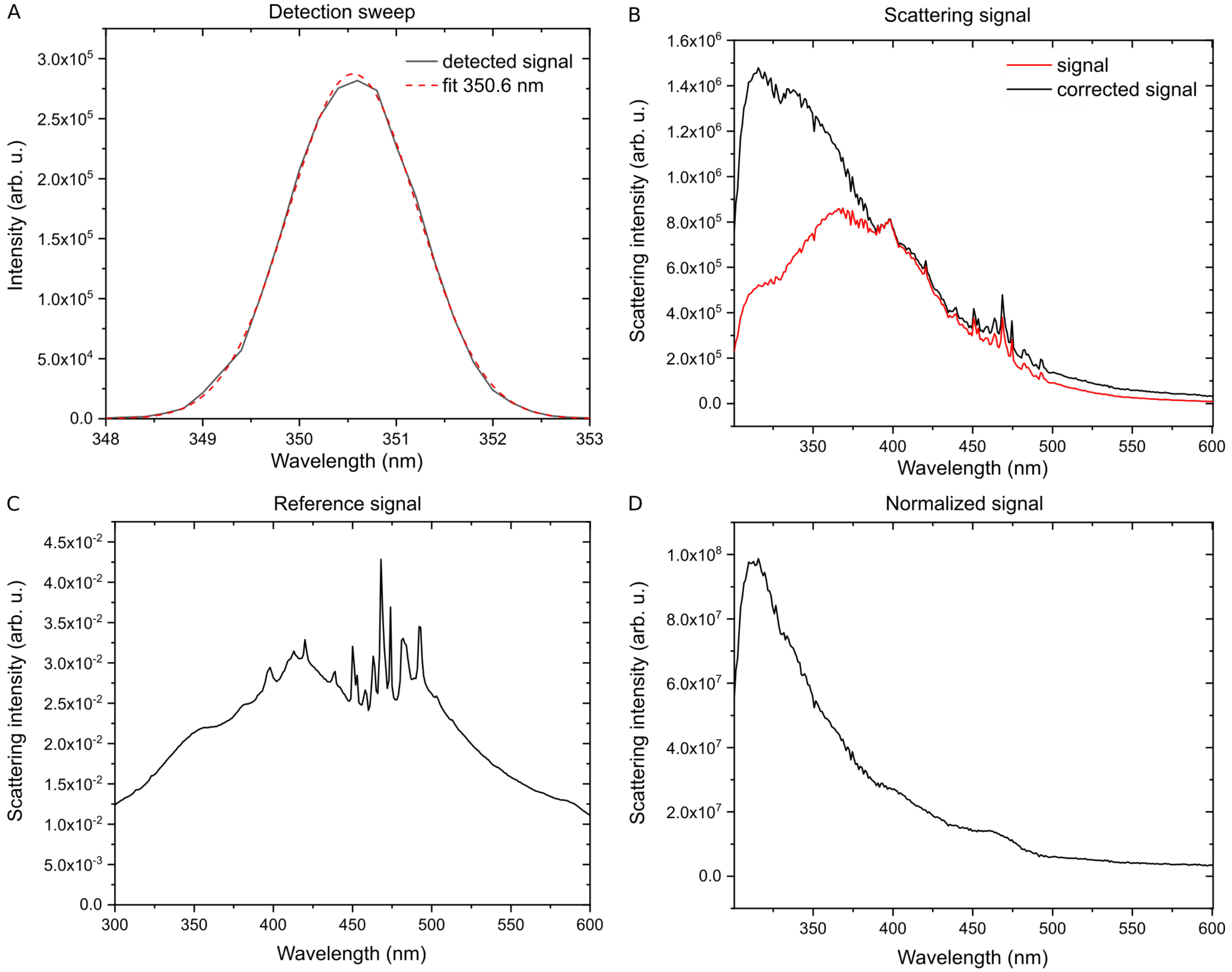

Supplementary Figure S 1. Detected scattered light intensity for different measurement configurations. (**A**) Offset of the detection path monochromator. This can be measured by setting a fixed excitation wavelength (here 350 nm) and doing a narrow sweep of the detection monochromator. The position of the measured scattering peak can then be used as the offset of the detection monochromator. (**B**) Wavelength dependence of the scattering intensity can be measured with a synchronous sweep of both the excitation and detection monochromators. The red curve shows the detected scattering signal at different wavelengths of excitation and detection. The black curve is the same signal, but corrected by a wavelength dependent factor that accounts for the wavelength-dependent sensitivity of the detector. We can see that the correction is most striking for wavelengths below 400 nm, as the photomultiplier used in our system is less sensitive in that range. The sharp peaks in the range 400–500 nm are caused by spectral lines of the light source. (**C**) Graph of the reference signal, *i.e.* the lamp intensity at different wavelengths. We can clearly see the spectral lines of the light source which caused additional peaks in (B). We can also see that the intensity of the lamp is diminished below 400 nm. (**D**) Corrected scattering signal normalized by the reference signal. We can see that after applying the wavelength-dependent corrections the scattering intensity is increasing with decreasing wavelengths. However, the strong scattering signal at short wavelengths is a consequence of the numerical corrections, so for better signal to noise ratio it is better to do the measurement at wavelengths where both the raw scattering signal in (B) and the lamp intensity in (C) are relatively high.

Note S2: Scattering intensities at different wavelengths during folding.

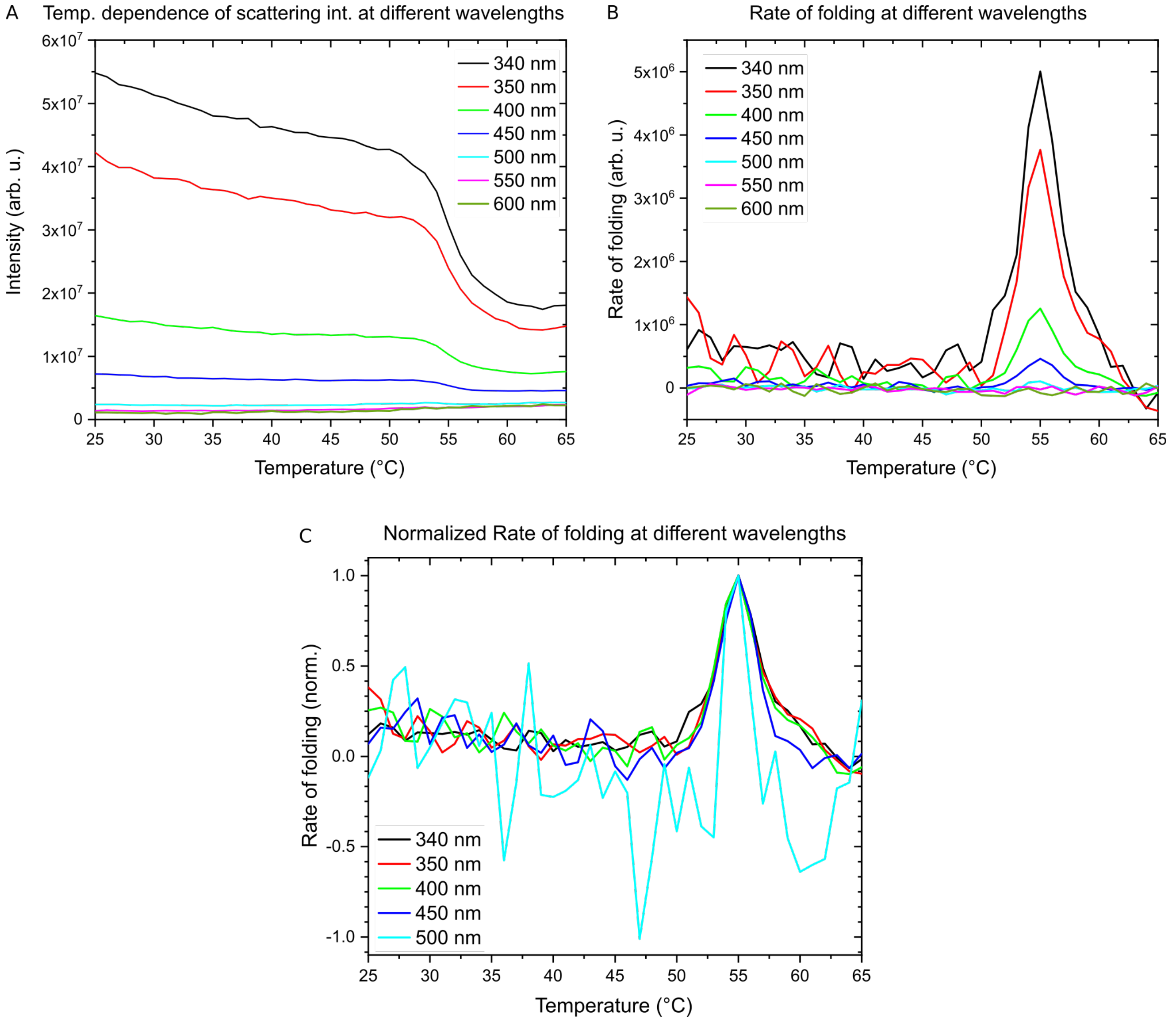

Supplementary Figure S 2. Folding of the tetrapod structure with a cooling rate of 20 min/℃. (**A**) Scattering intensity values at different wavelengths. (**B**) Temperature derivatives of the scattering intensities show that with increasing wavelength the scattering peak in the Rate-of-folding curves is becoming less and less pronounced. (**C**) The normalized Rate-of-folding curves (from (B)) show that the scattering peaks have a very similar shape for peaks at different wavelengths, but the signal-to-noise ration decreases, which can be compensated for by longer exposure times. Curves for wavelengths above 500 nm are omitted because of their poor signal-to-noise ratio.

Note S3: Background signals from buffer, staples and scaffold.

When monitoring folding and unfolding processes with techniques like qPCR and UV absorption measurements, the scaffold and the staples can produce considerable background signal. To check for this, we prepared different mixtures omitting one or more of the folding components and ran repeated heating and cooling cycles. Fig. S3A shows repeated cycles of heating and cooling for a cuvette filled only with a typical folding buffer. In Fig. S3B a similar measurement was done for a mixture of all the staples of the tetrapod structure in the same buffer and for Fig. S3C we used only the scaffold in buffer, at the same concentration as in a folding mixture. Out of the three measurements the highest signal comes as expected from the scaffold, as it has the highest molecular weight of around 2.5 MDa. None of the three samples show behaviour which would resemble the increase in intensity during folding of DNA origami (*i.e.* an increase in intensity during cooling of the sample typically in the 50 °C to 60 °C range and a decrease in intensity at a somewhat higher temperature while heating the sample), but they do exhibit an increase in intensity upon heating the sample above 60 °C. We do not have a conclusive answer to the cause of this increase, but we hypothesise it is at least partially due to formation of gas bubbles in the liquid because of the reduced solubility of gasses at high temperatures. This is supported by the observation that the high scattering intensity decreases over time if incubating at high temperature, probably because gas bubbles start to leave the liquid. We can see something similar when cycling the sample through several cycles of heating and cooling, where the maximum intensity at the highest temperature typically decreases with each cycle. It is possible that the scaffold somehow contributes to this high temperature increase as we have noticed that this effect tends to be stronger in samples with the scaffold.

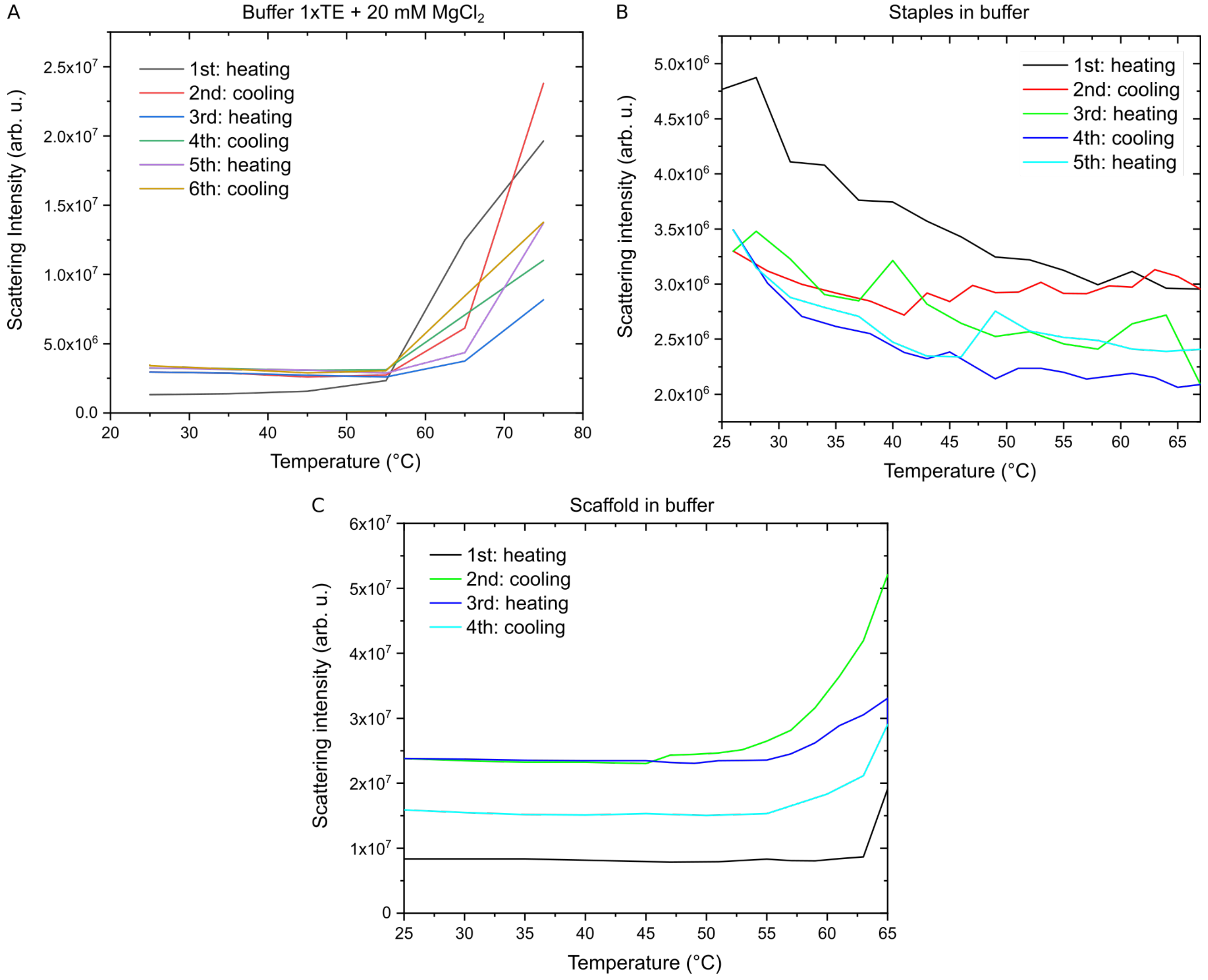

Supplementary Figure S 3. Background signals in a folding mixture. (**A**) Scattering intensity during 6 rounds of heating and cooling of a typical DNA origami folding buffer. (**B**) Scattering intensity during 5 rounds of heating and cooling of the tetrapod staples in a typical DNA origami folding buffer. (**C**) Scattering intensity during 4 rounds of heating and cooling a typical DNA origami scaffold at 10 nM in a typical DNA origami folding buffer.

Note S4: qPCR *vs.* light scattering.

For the qPCR measurements we prepared 100 μL of a folding mixture at 10 nM scaffold and 100 nM staple concentration in 1$\times$TE buffer with appropriate $MgCl_2$ concentration. Additionally we prepared 50 μL of two "blank" mixtures for each structure, where we would substitute either the scaffold or the staples with MilliQ water. All samples were stained with SyberSafe fluorescent intercalating dye at 4$\times$ concentration. We transferred the samples into an optical quality PCR tube strip in 25 μL aliqotes and ran three rounds of annealing and heating ramps on a Bio-Rad C1000 Touch with a CFX96 optical unit measuring the fluorescence at the end of each step. The measurements were then averaged between the different aliquotes and runs (for the folding mixture that means an average of 12 runs per structure) and the staple signal (which causes the most background) was subtracted from the full folding mixture. The rates of folding were obtained by taking the derivative of the averaged and subtracted measurements.

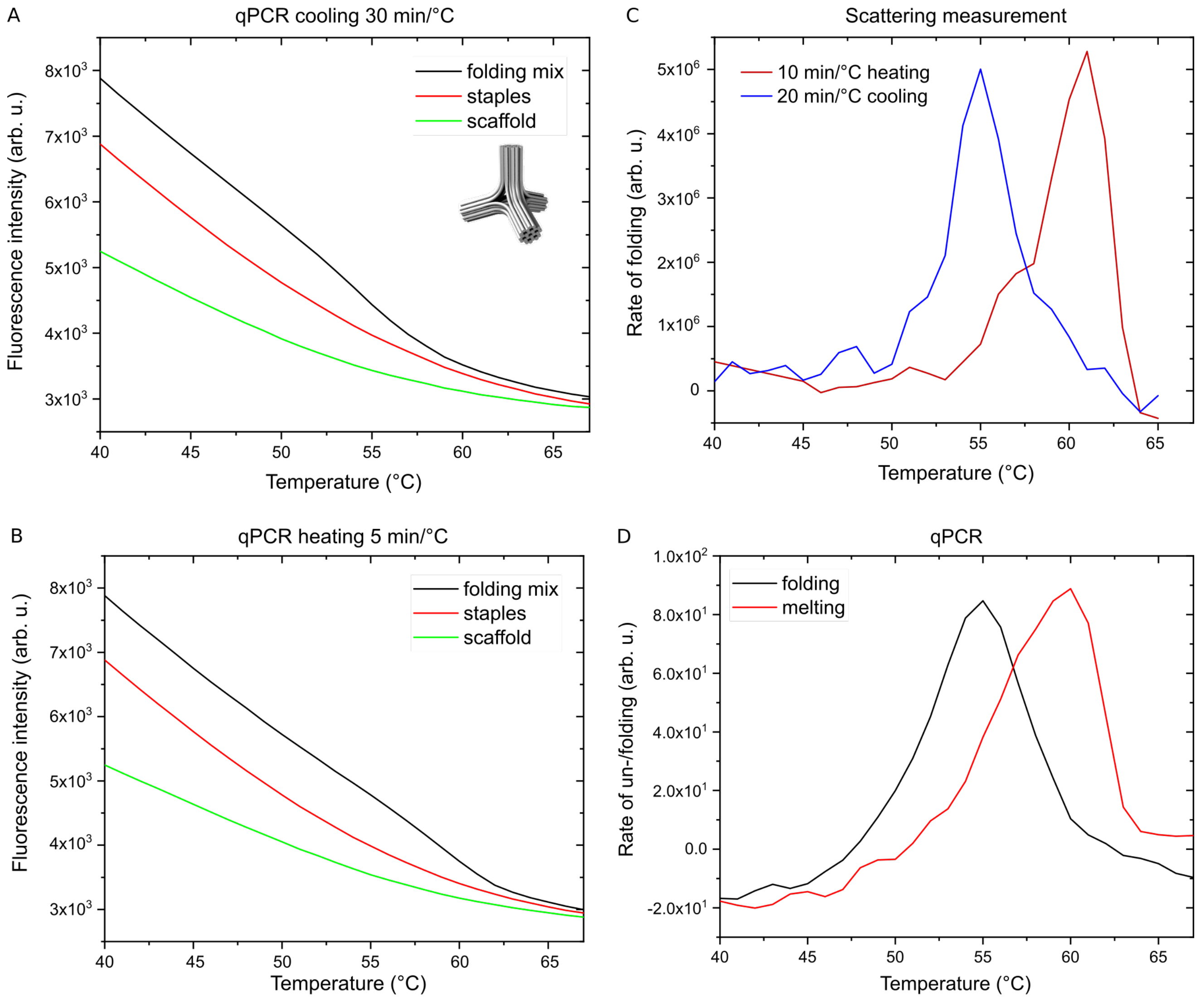

Supplementary Figure S 4. Folding of the "tetrapod" structure. (**A**) qPCR fluorescence intensity during cooling. (**B**) qPCR fluorescence intensity during heating. (**C**) Rate-of-folding and unfolding derived from scattering measurements. (**D**) Rate-of-folding and unfolding derived from qPCR measurements.

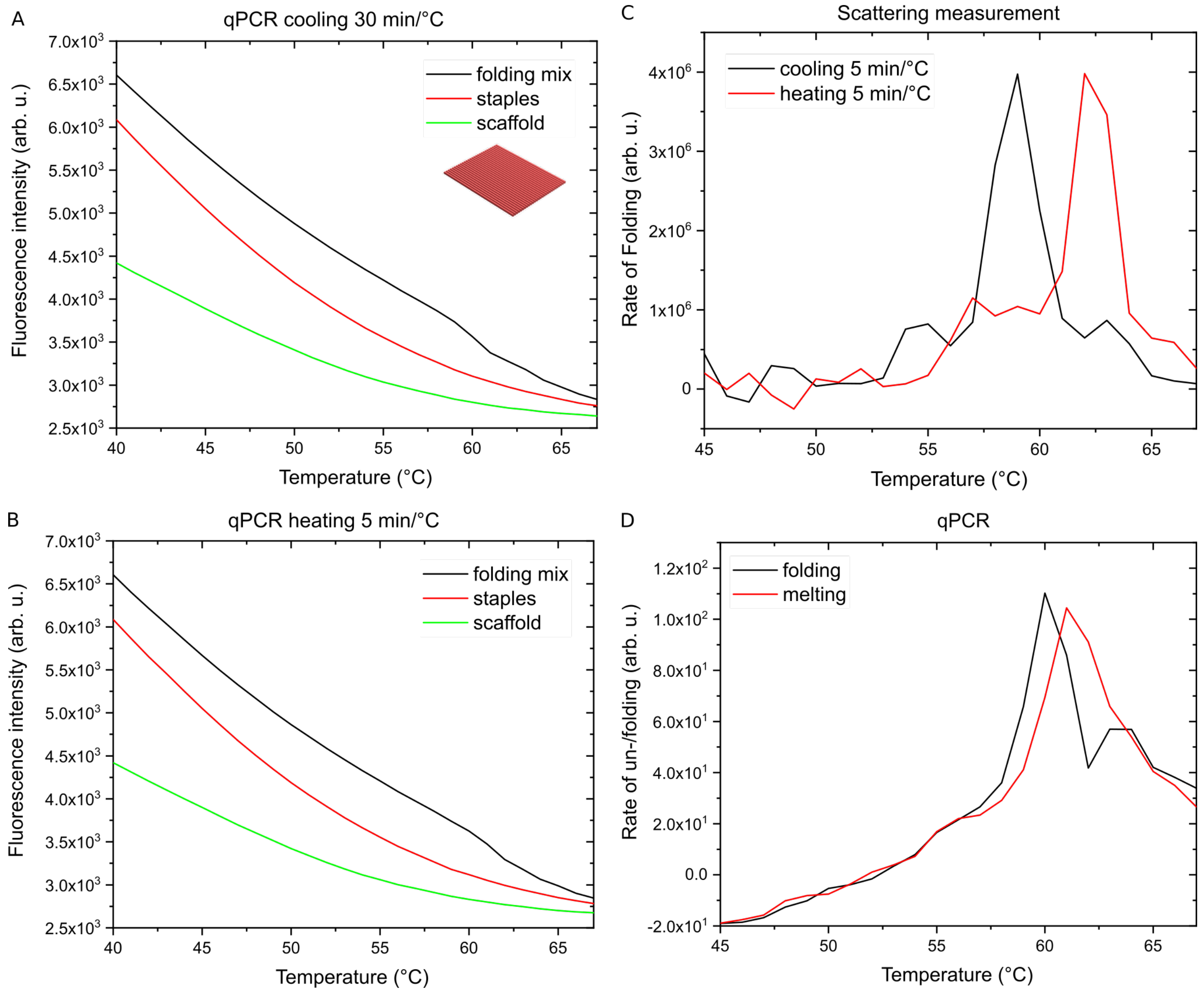

Supplementary Figure S 5. Folding of the RRO structure. (**A**) qPCR fluorescence intensity during cooling. (**B**) qPCR fluorescence intensity during heating. (**C**) Rate-of-folding and unfolding derived from scattering measurements. (**D**) Rate-of-folding and unfolding derived from qPCR measurements.

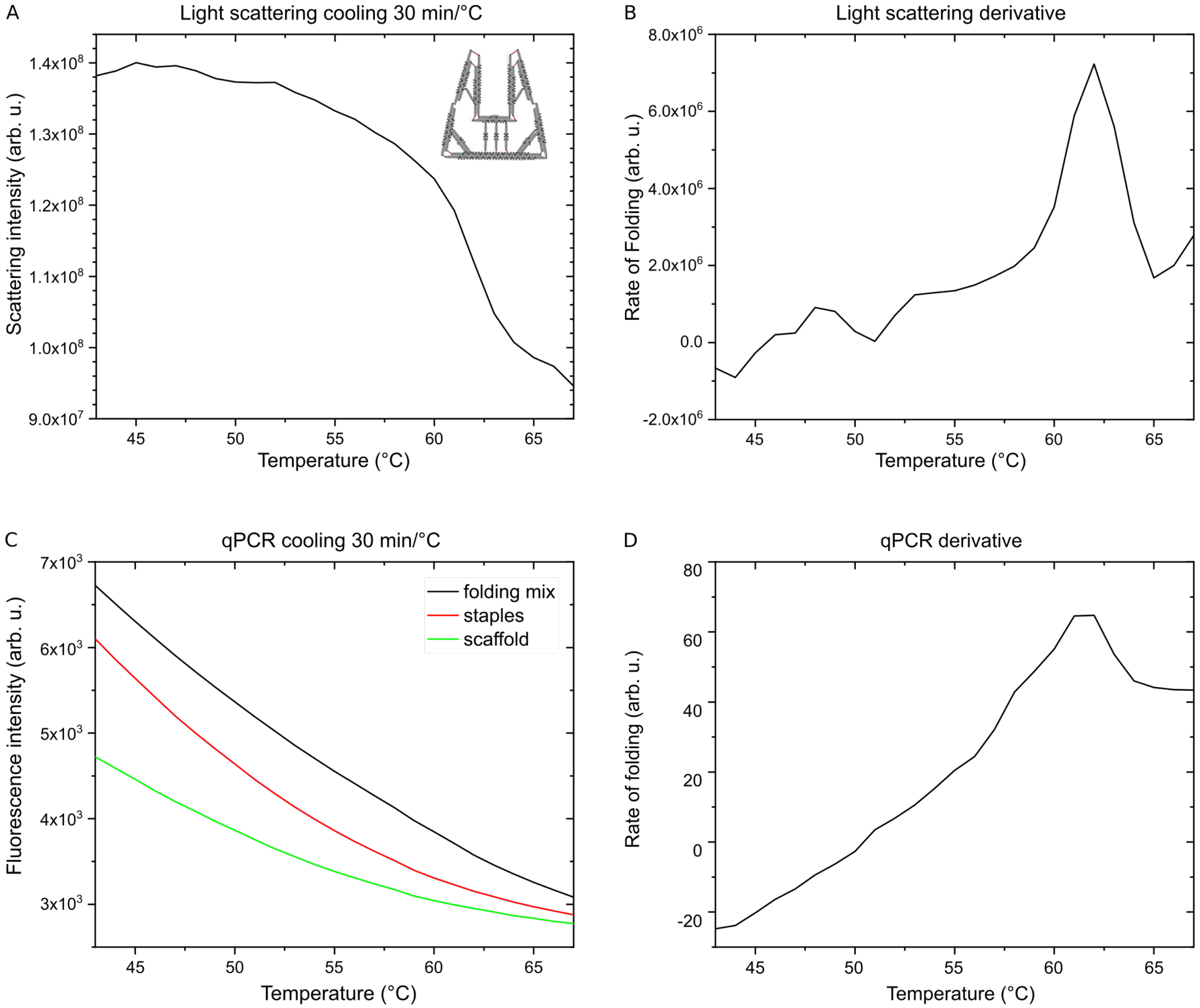

Supplementary Figure S 6. Folding of the "gripper" structure. (**A**) Scattering intensity during cooling. (**B**) Rate-of-folding derived from the scattering measurement. (**C**) qPCR fluorescence intensity during cooling. (**D**) Rate-of-folding derived from the qPCR measurements.

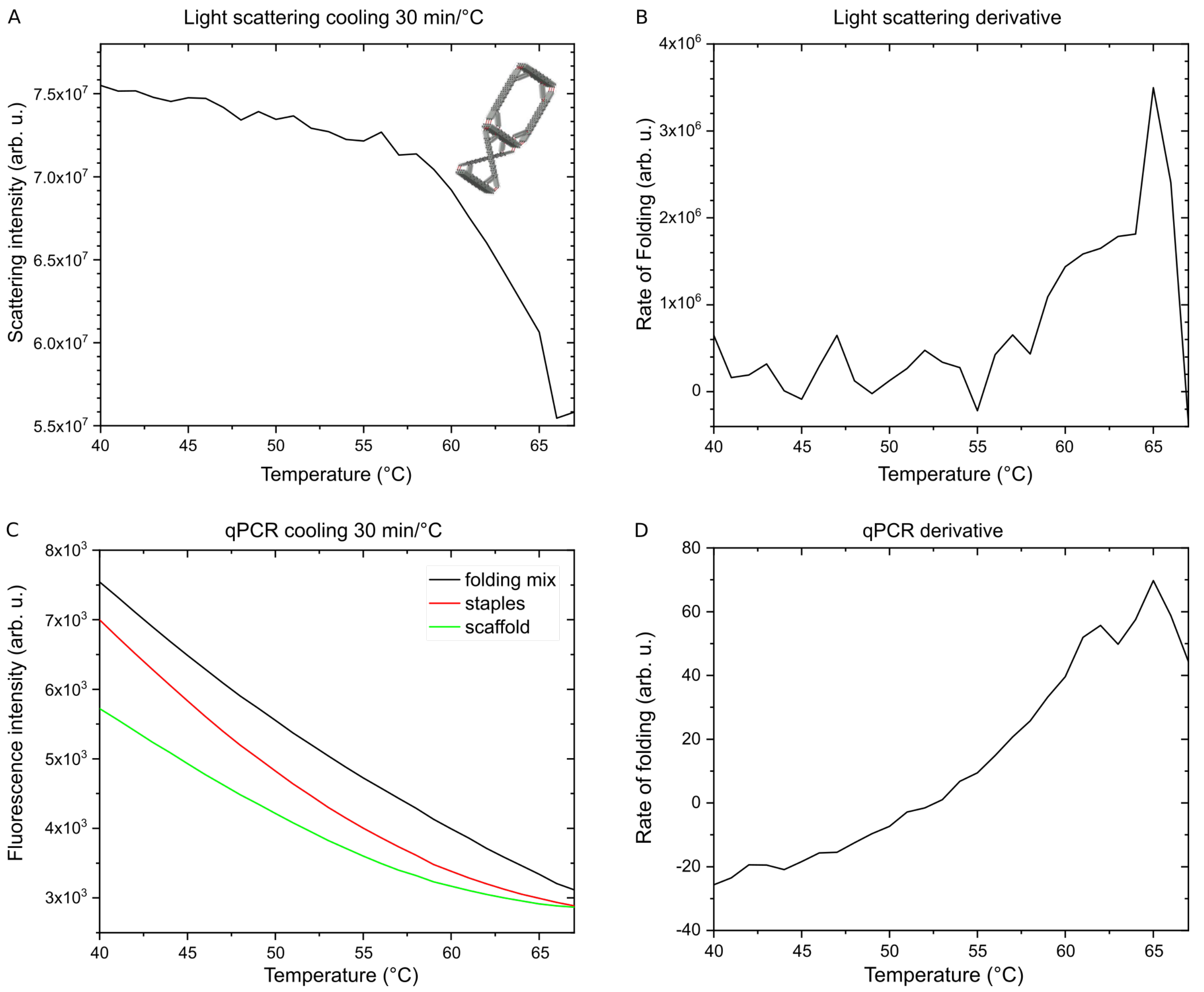

Supplementary Figure S 7. Folding of the "compliant compound joint" structure. (**A**) Scattering intensity during cooling. (**B**) Rate-of-folding derived from the scattering measurement. (**C**) qPCR fluorescence intensity during cooling. (**D**) Rate-of-folding derived from the qPCR measurements.

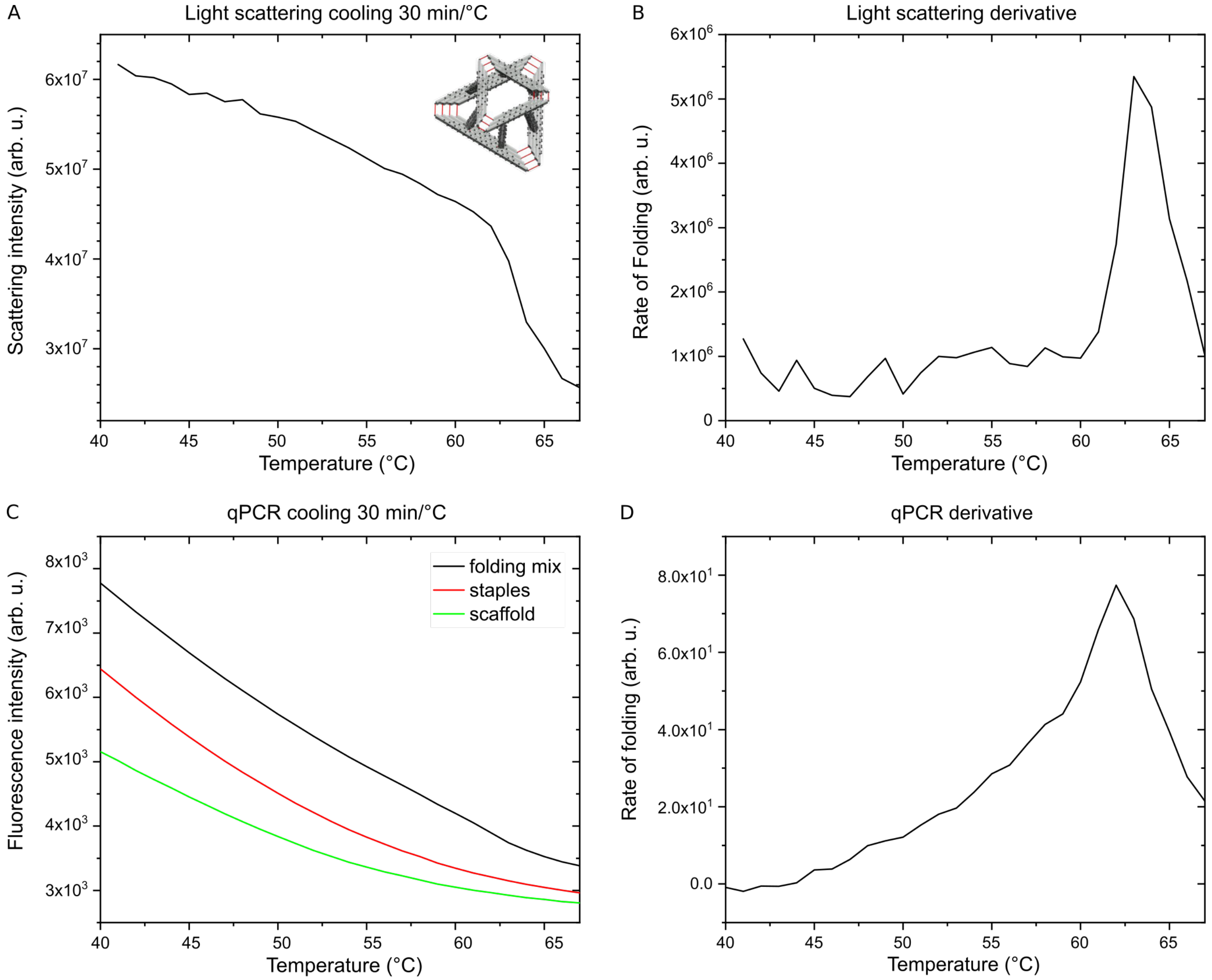

Supplementary Figure S 8. Folding of the "Stewart platform" structure. (**A**) Scattering intensity during cooling. (**B**) Rate-of-folding derived from the scattering measurement. (**C**) qPCR fluorescence intensity during cooling. (**D**) Rate-of-folding derived from the qPCR measurements.

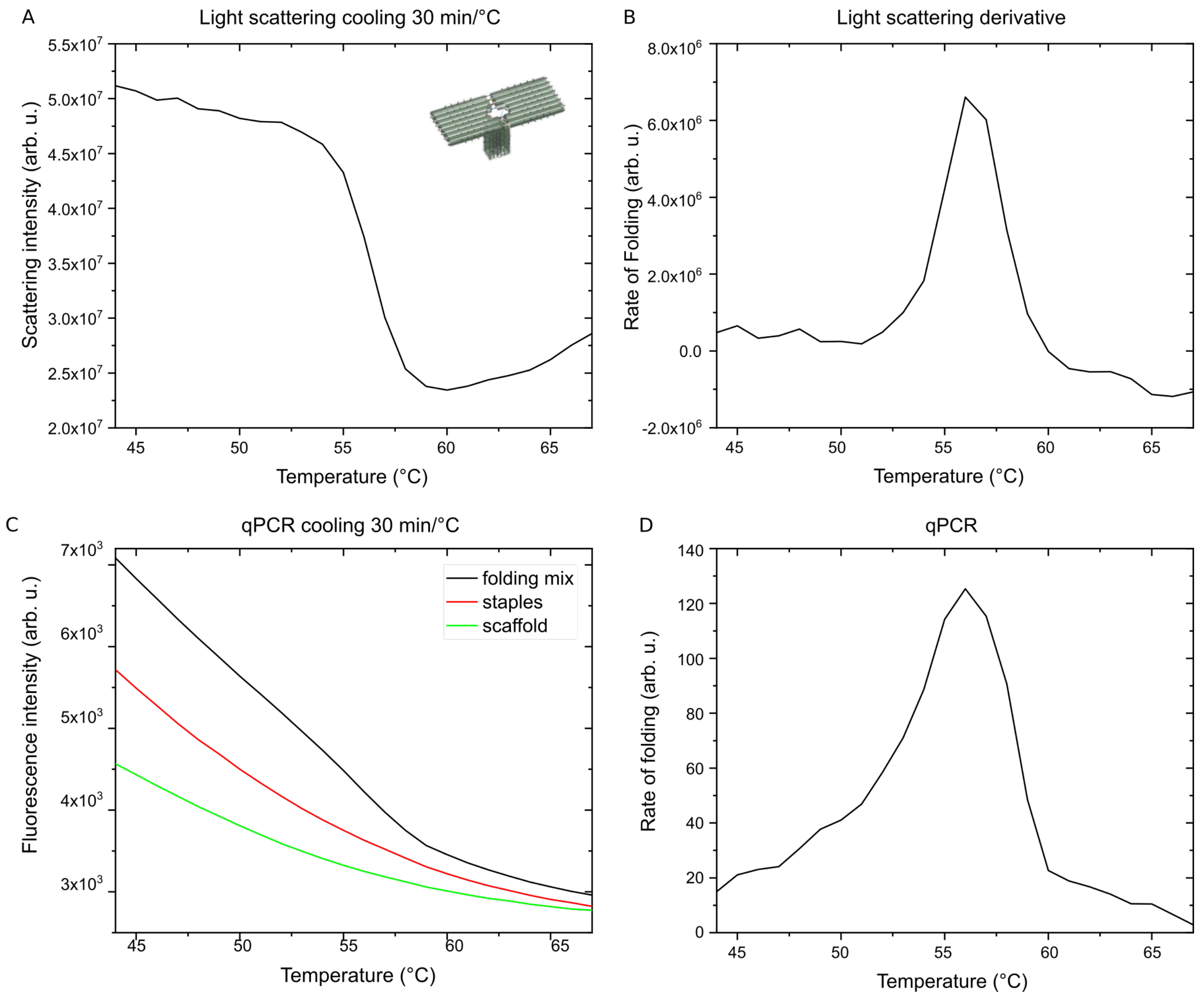

Supplementary Figure S 9. Folding of the "nanopore" structure. (**A**) Scattering intensity during cooling. (**B**) Rate-of-folding derived from the scattering measurement. (**C**) qPCR fluorescence intensity during cooling. (**D**) Rate-of-folding derived from the qPCR measurements.

Note S5: Non-normalized scattering intensities during DNase I digestion.

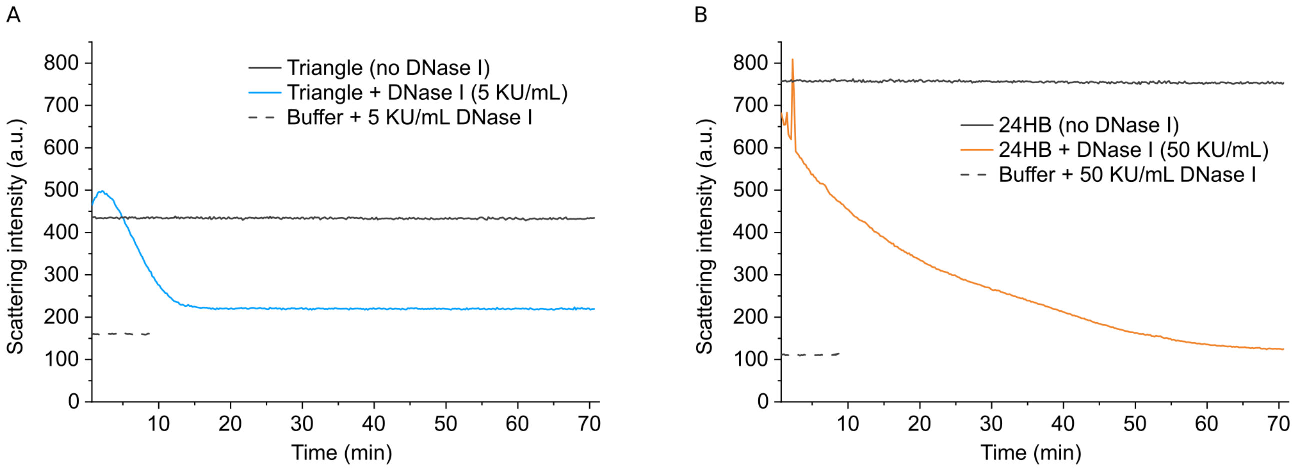

Supplementary Figure S 10. Scattering intensity values at 340 nm for RTO and 24HB origami structures, as presented in the Figure 5 in the main article, but without background subtraction and intensity normalization. The intensity values in panel A have been obtained with a PMT voltage of 650 V, while a 620 V voltage was used for the experiment in panel B to avoid saturating the detector when measuring the more strongly scattering 24HB. The intensity values thus cannot be directly compared between panels A and B. (**A**) The digestion of 4 nmol/L RTO in the presence of 5 KU/mL DNase I. The digestion curve is compared both to a sample with 4 nmol/L RTO without DNase I, and to a reference sample containing the digestion buffer and 5 KU/mL DNase I. (**B**) The digestion of 4 nmol/l 24HB with similar reference data sets as for the RTO.
