# Supplementary material for: A label-free light-scattering method to resolve assembly and disassembly of DNA nanostructures
Source: Biophys J. 2022 Oct 29;121(24):4800–9. doi: 10.1016/j.bpj.2022.10.036 (PMC9811603; doi:10.1016/j.bpj.2022.10.036)
Supplement: Document S2. Article plus supporting material [file mmc2.pdf]

Biophysical Journal
Article

A label-free light-scattering method to resolve assembly and disassembly of DNA nanostructures

Heini Ijäs,[1,2] Tim Liedl,[2] Veikko Linko,[1,3,*] and Gregor Posnjak[2,*]
[1]Biohybrid Materials, Department of Bioproducts and Biosystems, Aalto University, Aalto, Finland; [2]Faculty of Physics and Center for NanoScience (CeNS), Ludwig-Maximilians-University, Munich, Germany; and [3]LIBER Center of Excellence, Aalto University, Aalto, Finland

ABSTRACT DNA self-assembly, and in particular DNA origami, has evolved into a reliable workhorse for organizing organic and inorganic materials with nanometer precision and with exactly controlled stoichiometry. To ensure the intended performance of a given DNA structure, it is beneficial to determine its folding temperature, which in turn yields the best possible assembly of all DNA strands. Here, we show that temperature-controlled sample holders and standard fluorescence spectrometers or dynamic light-scattering setups in a static light-scattering configuration allow for monitoring the assembly progress in real time. With this robust label-free technique, we determine the folding and melting temperatures of a set of different DNA origami structures without the need for more tedious protocols. In addition, we use the method to follow digestion of DNA structures in the presence of DNase I and find strikingly different resistances toward enzymatic degradation depending on the structural design of the DNA object.

SIGNIFICANCE We introduce a user-friendly technique to unravel DNA origami folding and melting processes in real time based on light scattering. Unlike other existing techniques, our method is simple, label-free and fast, and it does not require specific equipment or tedious control experiments. DNA origami assembly processes can be performed and resolved using common strand concentrations and reaction volumes, thus allowing the further use of the analyzed samples.

INTRODUCTION

Starting with its introduction by Ned Seeman in 1982 (1), DNA self-assembly by design has developed from an academic discipline into a mature and application-oriented field of research that is commonly referred to today as DNA nanotechnology (2). Researchers in this field address questions ranging from cellular biology (3) and nanomedicine (4,5) to material sciences (6).

One of the important steps in the process of establishing DNA nanostructures as reliable tools in the various disciplines was the invention of two- and three-dimensional DNA origami (7–10), where a long, phage-derived single-strand DNA (ssDNA) is folded into a desired shape by a set of oligonucleotides. The robustness of this method and the quick development of easy-to-use software tools (11) have lowered the barrier for newcomers to enter with little to no DNA chemistry knowledge (12). Using DNA origami and other DNA self-assembly schemes, it is now possible to design and assemble structures of almost arbitrary shapes with nanometer resolution. Such designer DNA structures can be further modified by attaching proteins, dyes, or inorganic nanoparticles, and they can dynamically switch between different configurations (13,14). This unique set of properties has enabled researchers to realize a plethora of potential drug-delivery (15) and gene-editing tools (16) and biosensing and plasmonic devices (17–20), as well as larger structures like three-dimensional crystalline lattices (21), micron-sized capsids (22), finite two-dimensional structures (23), and macroscopic ordered assemblies (24).

Critical to all applications, however, is a high degree of control over the assembly process. In fact, testing a newly designed structure in the lab requires parameter optimization. The success of DNA nanostructure folding at different concentrations of positively charged ions, which mitigate the interaction between the negatively charged backbone "scaffold" ssDNA strand and the equally charged "staple"

Submitted June 15, 2022, and accepted for publication October 24, 2022.

*Correspondence: veikko.linko@aalto.fi or gregor.posnjak@physik.uni-muenchen.de

Veikko Linko's present address is Institute of Technology, University of Tartu, Estonia

Editor: Alberto Perez.

https://doi.org/10.1016/j.bpj.2022.10.036

© 2022 Biophysical Society.

This is an open access article under the CC BY license (http://creativecommons.org/licenses/by/4.0/).

oligonucleotides, is easily and routinely monitored by agarose gel electrophoresis (AGE). Another important parameter, however, the temperature-controlled annealing ramp, usually receives much less attention. Typically, the annealing process starts with a short high-temperature step, depending on the laboratory culture, anywhere between 65°C and 95°C. This step serves to denature all previously formed duplexes into single strands. This is followed by a sequence of longer steps with gradually decreasing temperature until room temperature is reached. The temperature, duration, and number of annealing steps can be optimized, but this either takes an impractical amount of trial-and-error iterations or a method capable of monitoring the folding.

Several methods have been reported to examine the folding process of DNA structures. Observation of optical extinction of a DNA folding solution at 260 nm is a relatively straightforward option, where the decrease of absorbance indicates the formation of DNA duplexes (hypochromicity) (25). This approach may work well for the simple duplex-DNA forming solutions, but DNA origami folding solutions typically contain 5–10× excess of staple strands to ensure high folding yields, which means that only a small portion of the DNA is hybridized during folding. Because of that, the absorbance decrease upon the formation of duplex domains is obscured by the "background" absorption of the unhybridized staple strands. It is possible to observe the process either by analyzing the possible folding pathways (26), slowing the assembly down and using high-speed atomic force microscopy (AFM) (27), or by stopping or "freezing" the folding, followed by downstream analysis such as transmission electron microscopy (TEM) and AFM imaging (28–31).

The methods reported so far that are capable of monitoring DNA origami folding or melting in situ rely on special equipment, such as small-angle X-ray scattering (32), or involve tedious and costly labeling processes (33–35) and process optimization (27,28). For example, it is possible to dope the folding solution with an intercalating dye, which shows enhanced fluorescence when it is incorporated into the double-stranded DNA (dsDNA) domains. If the fluorescence of such a dye is determined during the folding of a DNA origami structure, the changes in fluorescence signal can be used as a measure of the rate of hybridization of ssDNA to dsDNA forms and, consequently, of folding into the desired structure (28). This approach requires an instrument capable of measuring fluorescence while changing the temperature of the sample from around 65°C to room temperature. Typically, this is carried out using quantitative polymerase chain reaction (qPCR) machinery, which is common but not ubiquitous. Another limiting factor is that the observed increase of dye fluorescence can be subtle compared with other parameters, affecting the fluorescence of the samples. Hence, several measurements for averaging and subtracting background signals are usually required.

Here, we present an alternative, label-free, easy, and robust approach to monitoring DNA origami folding, which is based on light scattering (Fig. 1). Both individual DNA molecules and folded DNA origami structures are much smaller than the wavelength of visible light. Light scattering from such objects takes place within the Rayleigh regime, where the intensity is strongly dependent on the size of the scatterer, leading to a clear difference in signals when comparing unfolded versus folded structures. Hence, we can deduce the state of folding of a DNA nanostructure by recording the scattering intensity of the folding solution while varying its temperature or degrading the nanostructures, e.g., digesting them with enzymes (Fig. 1).

Three main features of this method simplify the determination of DNA origami folding behavior: 1) the sample can be used as is, i.e., no labeling or intercalating dye is needed; the latter may potentially influence DNA hybridization. 2) The signal-to-noise ratio is so high that data interpretation does not rely on subtraction of background signals or other delicate control experiments. 3) The experiments can be performed using fluorescence spectrometers or dynamic-light-scattering equipment, which is common to many laboratories.

MATERIALS AND METHODS

The folding measurements were conducted on a Horiba Fluorolog with a heating stage and the DNase I measurements on an Agilent Cary Eclipse fluorescence spectrometer. The static light scattering (SLS) measurements were performed with a Wyatt DynaPro NanoStar using a 658 nm laser source and 90° scattering geometry. Both fluorescence spectrometers use broad-spectrum lamps (Hg in the case of Horiba Fluorolog and a Xenon flashlamp in the Agilent Cary Eclipse) with a monochromator for selection of the excitation wavelength. The light that scatters on the sample is collected at a 90° angle with respect to the incoming beam to avoid detection of the excitation light. The scattered light passes through the second monochromator for selection of a detected wavelength followed by the detection photomultiplier. Most samples can have fluorescent signals, but they are typically much smaller than the scattering signals—at least an order of magnitude, and often more than three orders of magnitude. They can be easily separated from the scattering signals as they are Stokes shifted to longer wavelengths. To check for this, one can fix the excitation wavelength and sweep the detection wavelengths so they also include the excitation wavelength.

Because Rayleigh scattering is elastic, the scattered light has the same wavelength as the incoming light. To measure it, both monochromators must transmit the same wavelength. Typically, there is a small offset between the two because of alignment imprecision. To compensate for this, the excitation monochromator can be set to a fixed wavelength, and the detection monochromator can be scanned around this wavelength to determine the offset between the two (Fig. S1). After taking this offset into account, both excitation and detection monochromators can be scanned to measure the wavelength dependence of scattering as shown in Fig. 2 *A*. To monitor the changes of scattering intensity in DNA origami, it is enough to use a single wavelength (typically a 1 nm band of wavelengths) for excitation and detection of the scattered signal at the same wavelength. This way of measuring is also suitable for measuring time series. When using a single wavelength for the scattering measurement, it is usually best to choose a wavelength at which the light source intensity, the detector sensitivity, and the detected signal are all relatively high (Fig. S1). In principle,

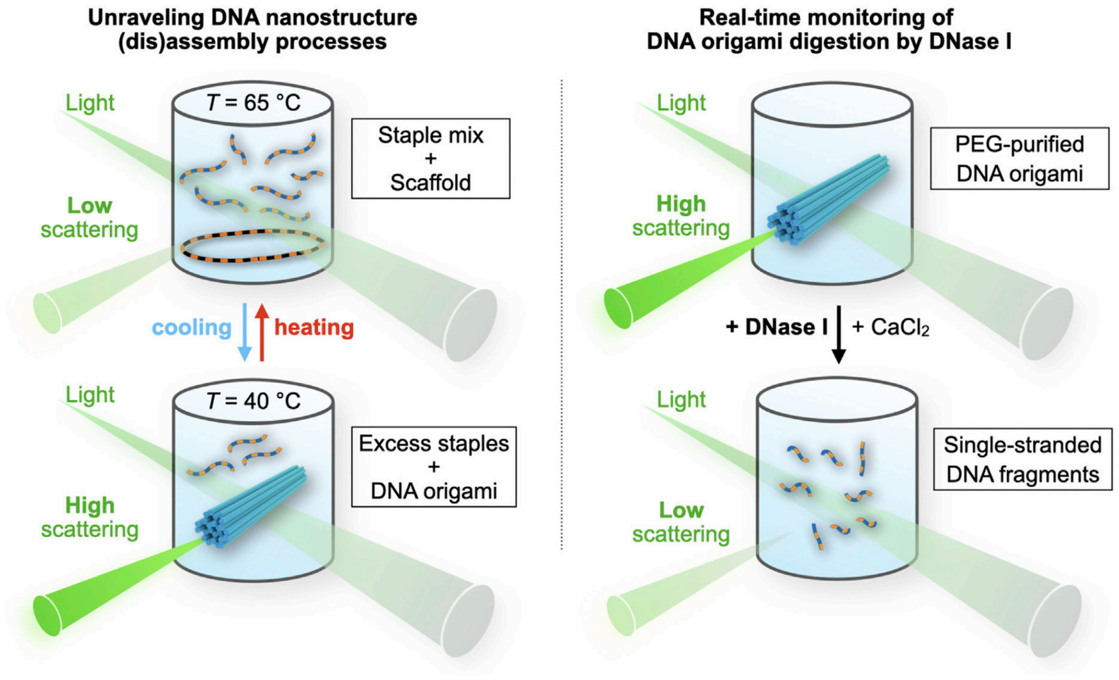

FIGURE 1 Schematic representation and demonstrated applications of the method. Left: a reaction mixture containing DNA nanostructure components is exposed with a monochromatic light (from, e.g., a fluorometer) and the scattered light intensity is recorded. When the solution temperature is varied, the changes in the scattering intensity reveal assembly/disassembly kinetics of these structures. Right: the method can be equally used for analyzing enzymatic degradation of the folded DNA origami nanostructures, as the ability to scatter incoming light is related to the intactness of DNA structures. To see this figure in color, go online.

the same information about the folding of the origami can be obtained with a longer wavelength (Fig. S2), but the lower signal-to-noise ratio needs to be compensated for with a longer exposure time.

DNA origami folding mixtures were prepared at a 10 nM scaffold concentration and a 100 nM staple concentration, in a 1× TE buffer (10 mM Tris with 1 mM EDTA adjusted to pH = 8 with HCl) supplemented with 12–24 mM $MgCl_2$ depending on the structure. The amount of sample needed for a measurement depended on the size of the cuvette: 250 μL for a 2 × 10 mm quartz cuvette (Hellma, Plainville, NY, USA) with clear sides or 20 μL for a disposable cuvette used for SLS measurements. Total acquisition times for each data point were typically 0.1 s for fluorometer setups and 50 s for SLS setups. Structures folded with optimized folding protocols were analyzed by AGE in 1% agarose gel stained with SyberSafe (Thermo Fisher Scientific, Waltham, MA, USA) at 1× concentration and run in an ice bath for 60 min at 80 V with a running buffer containing 1× TAE (40 mM Tris, 20 mM acetic acid, and 1 mM EDTA adjusted to pH = 8.0 with HCl) and 11 mM $MgCl_2$. Samples for TEM analysis were extracted from the gel by cutting and squeezing the bands of interest and incubating 5 μL of sample on an Ar glow-discharged TEM grid (formvar/carbon, 300 mesh Cu; Ted Pella, Redding, CA, USA) for 3 min. The samples were wicked off, washed once, and then stained for 10 s with 5 μL 2% uranyl formate. After drying the samples, the grids were imaged on a JEOL JEM1011 TEM operated at 80 kV.

For the DNase I digestion experiments, the Rothemund triangle (RTO) (7) and the 24-helix bundle (24HB) (36) DNA origami structures were thermally annealed according to the protocols described in the original publications at 20 nM scale. After annealing, the structures were purified of excess staple strands using polyethylene glycol-based precipitation (37). For the DNase I digestion, the purified origami structures were diluted to a final 4 nM concentration in a digestion buffer containing 0.2× TAE, 20 mM $MgCl_2$, 1 mM NaCl, and 1 mM $CaCl_2$. A solution of DNase I from bovine pancreas (Sigma-Aldrich, Burlington, MA, USA) was prepared in deionized water at 2,000 KU/mL and mixed into the DNA origami solution at either a 5 (RTO) or 50 KU/mL (24HB) final concentration. Immediately after adding the DNase I, the samples were transferred into a 2 × 10 mm quartz fluorescence cuvette (Hellma Analytics), and the DNase I digestion was monitored with a kinetic light-scattering measurement. The excitation and detection wavelengths were set at 340 nm, with both excitation and emission slits at 10 nm. Scattering intensities were collected at 0.2 min intervals over 70 min for both structures. The measurements were performed at room temperature.

As a reference for intact origami, the scattering measurement was performed for a sample of DNA origami without DNase I, where distilled water was added in a volume equal to the volume of DNase I in the digestion sample. A buffer baseline was obtained by measuring the scattering intensity from the digestion buffer supplemented with either 5 (for the RTO) or 50 KU/mL (for the 24HB) DNase I.

AGE analysis of the digestion was carried out in parallel to the scattering measurement: samples were taken from the digestion reaction mixture at specified time points and mixed with 1% sodium dodecyl sulfate (SDS) (Sigma-Aldrich) to a final SDS concentration of 0.1% to inactivate the DNase I. A 2% agarose gel was cast and stained with ethidium bromide (EtBr) (0.5 mg/mL). For loading the samples on an agarose gel after the digestion had been completed, each DNA origami-SDS sample was mixed with a 40% sucrose solution to a final sucrose concentration of 6.7%. The gel was run at 90 V for 45 min in a running buffer containing 1× TAE and 11 mM $MgCl_2$. Different folding protocols of DNA origami structures were tested by first folding the structures in a thermocycler and then running AGE gels (1% agarose with 1× TAE and 11 mM MgCl, stained with 1× SyberSafe, ran at 80 V for 1 h) to check for quality of folding and amount of aggregation.

RESULTS AND DISCUSSION

The intensity of Rayleigh scattering depends on the wavelength of scattering light, the sample concentration, and

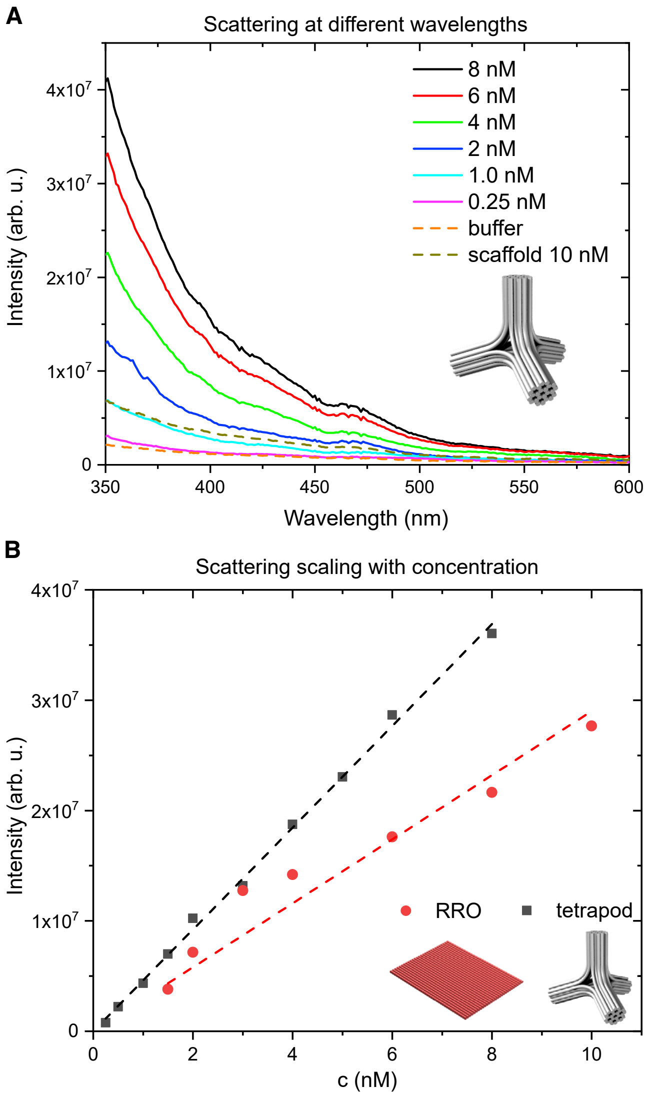

FIGURE 2 Wavelength and concentration dependence of light scattering on DNA origami. (*A*) Intensity of scattered light versus wavelength of excitation light for the tetrapod structure at different concentrations. (*B*) Concentration scaling of scattering intensity at a single excitation wavelength ($\lambda = 350$ nm) for the Rothemund rectangular origami (*left inset*) and the tetrapod (*right inset*). To see this figure in color, go online.

the molecular mass of the sample. If the excitation and detection monochromators of a fluorometer setup are set to the same wavelength, and this wavelength is scanned over the visible range, we can see how the intensity of scattered light changes for different wavelengths (Fig. 2 *A*). The scattering intensity in the Rayleigh regime is strongly wavelength dependent, but we did not observe the typical $1/\lambda^4$ dependence. We attribute this at least partially to the alignment and calibration of the instrument. We will later demonstrate that for monitoring the folding process, it is enough to follow the changes of the scattering intensity at a single wavelength, and importantly, the exact wavelength dependence of scattering does not play a role.

Fig. 2 *A* also shows that the scattering from a typical concentration of DNA origami is much stronger than the scattering from the buffer (10 mM Tris, 1 mM EDTA, and 11 mM $MgCl_2$). Scattering intensity at a fixed wavelength scales linearly with an increasing concentration of DNA origami as shown in Fig. 2 *B*. The scaling constant depends on the shape and size of the DNA origami. A flat single-layered Rothemund rectangle (left inset in Fig. 2 *B*; lateral dimensions of 90 × 70 nm and thickness of 2 nm (7)) scatters much less than a bulkier, three-dimensional tetrapod structure (Fig. 2 *B*, right inset; four 35 nm long and 15 nm thick arms, the cross-section of each arm is a 24HB).

To monitor the folding or unfolding process of DNA origami, it is enough to measure the scattering intensity at a fixed excitation wavelength while slowly changing the temperature of the sample. In Fig. 3 *A* we can see how the scattering intensity changes with temperature for a folding mixture for the tetrapod origami at different cooling and heating rates. The cooling protocols start with a 10 min melting step at 65°C, followed by 1°C cooling steps. At the end of each step, the scattering intensity is measured for an excitation wavelength of 350 nm. The scattering intensity is relatively low at high temperatures, indicating an unfolded state of the origami structure. As the temperature is lowered, at first the intensity rises relatively slowly, followed by a sharp increase within a narrow temperature range, after which the slope of increase is again much flatter. We interpret this 2.5-fold increase in scattering intensity as the transition of the DNA from the single-stranded form into the more compact DNA nanostructure with dsDNA domains, where the incorporation of the staple oligonucleotides roughly doubles the molecular mass of each scaffold strand. This doubling of molecular mass alone can be expected to increase the scattering intensity relative to the scattering intensity from the unpaired scaffold, accompanied by possible effects arising from the particle geometry change during folding. In addition, the molecular mass of each individual staple in the folding mixture is multiple orders of magnitude smaller than that of the scaffold or the assembled origami, and thus the staple mixture has a minimal contribution to the scattering signal (Fig. S3). This lack of background signal from the staples is a particular advantage of the light-scattering method when studying DNA origami folding. For example, both UV absorbance measurements or the qPCR method will have a background signal arising from hybridization events between staples, which is further amplified by the molar excess of staples used for folding.

The scattering method allows us also to monitor the unfolding of structures. If the already folded sample is heated, the events are reversed compared with folding—at low temperatures, the scattering intensity is large, indicating a folded structure, but as the sample is being heated, the scattering intensity begins to decrease rapidly at a structure-specific temperature. There is a clear hysteresis between the cooling and heating curves, indicating that after folding,

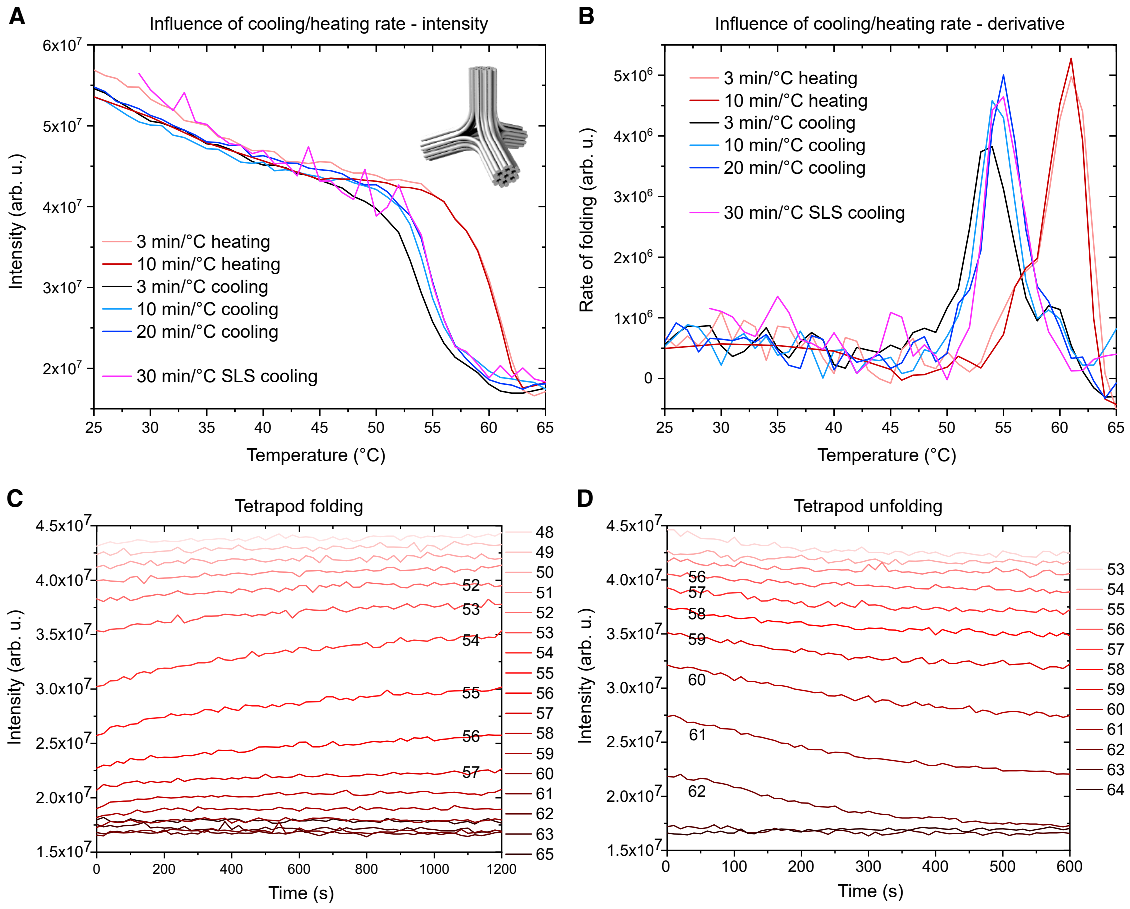

FIGURE 3 Scattering intensity of the folding mixture for the tetrapod structure (shown in *inset* in *A*) at different temperatures. (*A*) Temperature dependence of scattering intensity for different cooling and heating rates obtained with the fluorometer setup. An SLS measurement using a DLS setup is shown for comparison. (*B*) Derivatives of the curves shown in (*A*), i.e., the rates of folding (in arbitrary units). (*C* and *D*) Time series of scattering intensities at different temperatures during cooling (*C*) and heating (*D*). To see this figure in color, go online.

the structure as a whole unravels at higher temperatures than single sequences in the structure due to collaborative effects where many oligonucleotides collectively hold the long scaffold in the prescribed shape. The effective temperature range for folding or unfolding is even more apparent if we plot the derivative of the scattering intensity (Fig. 3 *B*). Measurements taken at different cooling rates reveal the dynamics of the folding process—the measurement with the fastest rate of cooling is shifted toward lower temperatures compared with measurements performed at slower rates, indicating that the folding process does not thermalize at such high rates of cooling, which might lead to kinetic traps during folding. The rate of heating has much less influence on unfolding of the structure, with the intensities for the two rates in the graph Fig. 3 *A* being almost identical. This indicates that unfolding is a much faster process than folding of the structure.

In Fig. 3, *A* and *B*, we show a measurement performed in the SLS mode of a dynamic-light-scattering instrument. The shape of the curve is very similar to those obtained with a fluorometer setup, but the data are much noisier. The SLS curve in Fig. 3 *A* is raw data multiplied only by a constant factor to match the amplitude of the other curves, but to calculate its derivative, we needed to apply a smoothing filter to the data set (SLS curve in Fig. 3 *B*). We attribute the noisiness of SLS data to the measurement being conducted with an excitation wavelength of 658 nm instead of shorter wavelengths. As can be seen in Fig. 2 *A*, the scattering signal is much weaker at this wavelength compared with the 350 nm we used in the fluorometer setup. Despite the noise, the increase in intensity during folding of the DNA origami is still large enough to discern which temperature range is the most important for successful folding of the structure.

During a typical DNA origami folding protocol, incubation steps at different temperatures do not have to be of equal lengths. Because of this, it is useful if the monitoring method offers information about the rate of folding at each temperature. One way of easily achieving this is to measure the scattering intensity in a time series at each temperature step. Fig. 3 *C* shows how the intensity changes over time for each temperature step. We can see that for many temperatures, there is only a small change in intensity at the

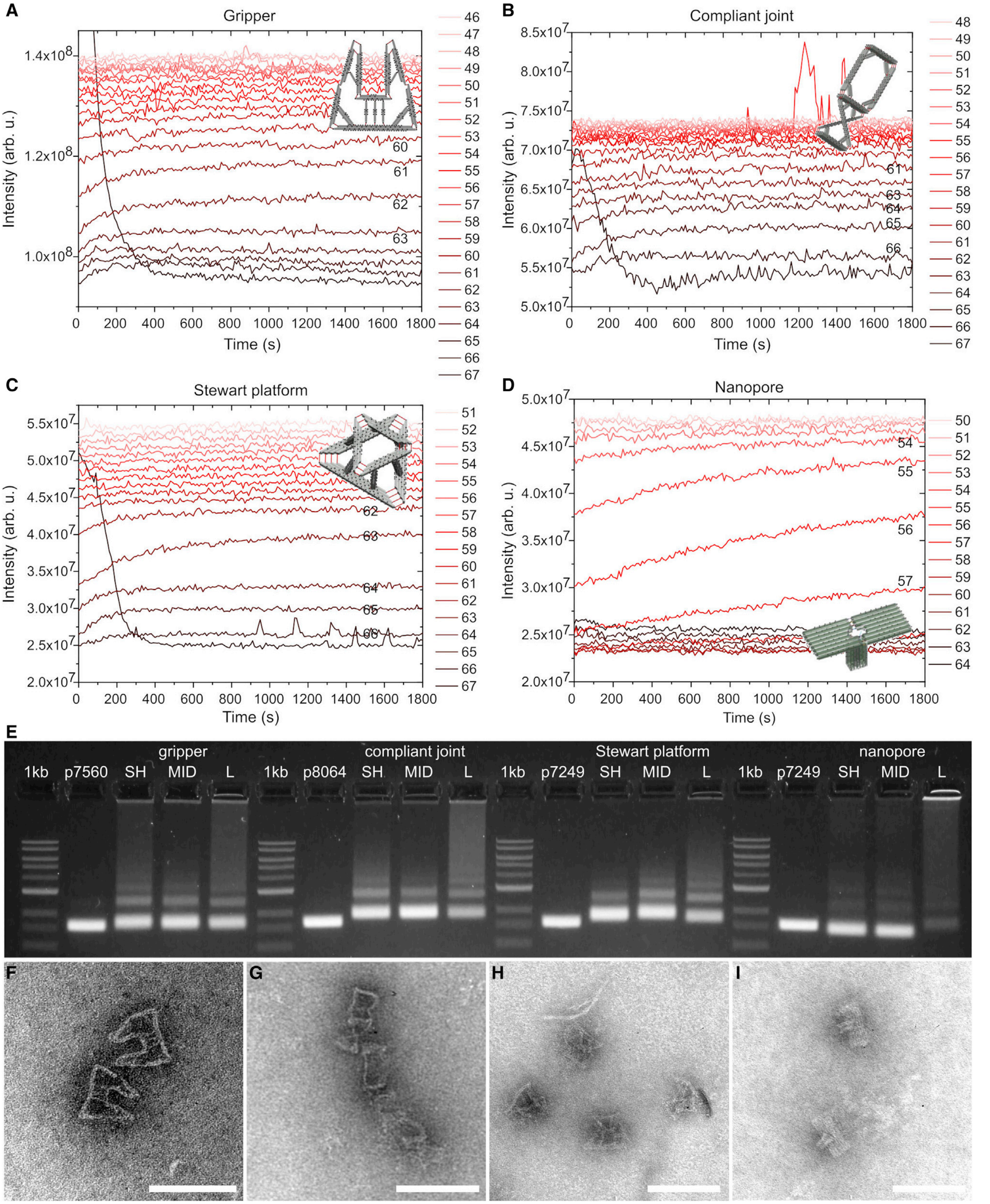

FIGURE 4 Examples of folding optimization for different structures. (*A–D*) Scattering-light-intensity change over time and at different temperatures for a gripper (*A*), a compliant joint (*B*), a Stewart platform (*C*), and a nanopore (*D*). The high scattering intensities at the beginning of the 67°C step are probably due to air bubbles (see Note S3). (*E*) AGE-based folding quality analysis for the same structures as in (*A*)–(*D*) using different thermal ramps. 1 kb is a DNA ladder reference, and p7249, p7560, and p8064 denote the different scaffold strand variants. For the folded structures, SH indicates a short 1–2 h folding

(legend continued on next page)

TABLE 1 Protocols used during folding optimisation

Structure[a]	Buffer	SH[b]	SH total time	MID[b]	Mid total time
Gripper	1 × TE + 22 mM $MgCl_2$	65°C–58°C: 10 min/°C	1 h 35 min	65°C–58°C: 30 min/°C	4 h 15 min
CCS	1 × TE + 18 mM $MgCl_2$	65°C–58°C: 10 min/°C	1 h 35 min	65°C–58°C: 30 min/°C	4 h 15 min
Stewart platform	1 × TE + 24 mM $MgCl_2$	63°C–61°C: 20 min/°C	1 h 15 min	63°C–61°C: 1 h/°C	3 h 15 min
Nanopore	1 × TE + 20 mM $MgCl_2$	57°C–55°C: 30 min/°C<br>54°C: 15 min/°C<br>53°C: 5 min/°C	2 h 5 min	57°C–55°C: 1 h/°C<br>54°C: 30 min/°C<br>53°C: 10 min/°C	3 h 55 min

CCS, compliant compound joint.

[a]The L protocol for all structures was the 2.5 day annealing protocol from (38): 65°C–61°C at 1 h/°C, 60°C–40°C at 2 h/°C, and 39°C–4°C at 30 min/°C.

[b]All annealing protocols start with a 15 min step at 67°C and stop with quick cooling to 4°C.

beginning of the step, with the intensity being constant the rest of the time, indicating that at that temperature, the folding process thermalizes quickly. At certain temperatures we can see the opposite—the intensity may keep increasing during most or even the whole step, indicating that due to the slow kinetics, the folding procedure could benefit from a longer annealing step at that particular temperature. In addition, we can extract similar information from the unfolding procedure while heating the sample (Fig. 3 *D*); however, as the heating leads to unraveled ssDNA molecules, the effect of kinetic traps is negligible, and the process is much faster.

To demonstrate the versatility of our method of monitoring the folding processes, we show how we can apply it to four structurally different DNA nanostructures, the designs of which were computer generated by the MagicDNA origami design tool (38). The "gripper" (Fig. 4 *A*) and the "compliant compound joint" (Fig. 4 *B*) are formed from several struts with different square lattice cross-sections, the "Stewart platform" (Fig. 4 *C*) with two triangular planes connected by 2 × 2 helix bundle square struts, and the "nanopore" (Fig. 4 *D*) with a flat platform using the hexagonal lattice and a hollow tower, which uses the square lattice. In the original publication, all four structures were reported to fold in a long 2.5 day program. The time series in Fig. 4 allows us to identify the temperatures at which the majority of the folding process happens. These particular temperature steps are characterized by significant increases of scattering intensity. The time-dependent measurement additionally enables the analysis of the kinetics of the folding process. For example, in the case of the first three structures (Fig. 4, *A–C*), most of the increase of the scattering intensities happens in the first 5 to 10 min at each relevant temperature, indicating relatively fast folding dynamics. In the case of the nanopore (Fig. 4 *D*), the scattering intensity at each relevant temperature increases slowly throughout the 30 min measurement interval, thus indicating a slow folding. Therefore, we use this information to derive shorter, optimized folding protocols, in which the cooling ramp starts around the first temperature with a large scattering intensity increase, and the cooling rate is adjusted to match the observed folding dynamics. The temperature steps of all the protocols are described in Table 1.

We analyzed the quality of folding of each structure and protocol by AGE (Fig. 4 *E*) and TEM of structures extracted from leading bands in the agarose gel (Fig. 4, *F–I*). We observed that for all structures, the three tested folding protocols result in almost identical gel bands, but the shorter, optimized protocols produce many fewer multimers and aggregates compared with the 2.5 day protocol. This suggests that prolonged incubation of folded origami in the folding mixture at elevated temperatures can significantly contribute to multimerization and aggregation of origami structures, and therefore it is desirable to exclude these temperatures from the folding protocol.

In addition to the folding and melting over temperature ramps, light scattering can also be applied to other types of DNA origami assembly and disassembly processes taking place in solution. To demonstrate this, we subject two different DNA origami structures—the RTO and a 24HB—to enzymatic digestion by DNase I and follow the processes in real time with light scattering. RTO and 24HB designs are chosen for this study because they are known to differ greatly in their structural stability under DNase I digestion (36). During the enzymatic digestion, the origami structures are degraded into short dsDNA and ssDNA fragments, which scatter a negligible amount of the 340 nm excitation light compared with the intact structures. As seen in Fig. 5 *A*, the degradation of the origami structures can be easily followed by the decrease of the intensity of scattered light. The notably higher DNase I resistance of the 24HB is clearly visible in the slower decay of the scattering intensity when compared with the RTO. Even in the presence of a 10-fold DNase I concentration, the 24HBs require approximately six times longer digestion time than the RTOs before the scattering intensity drops to the baseline level, indicating a complete degradation.

protocol, MID, ~4 h folding; L, a long 2.5 day program. Details of the folding protocols are given in Table 1. (*F–I*) Transmission electron microscopy images of structures extracted from the leading gel band of the MID length folding for (*F*) gripper, (*G*) compliant joint, (*H*) Stewart platform, and (*I*) nanopore. All scale bars are 100 nm. To see this figure in color, go online.

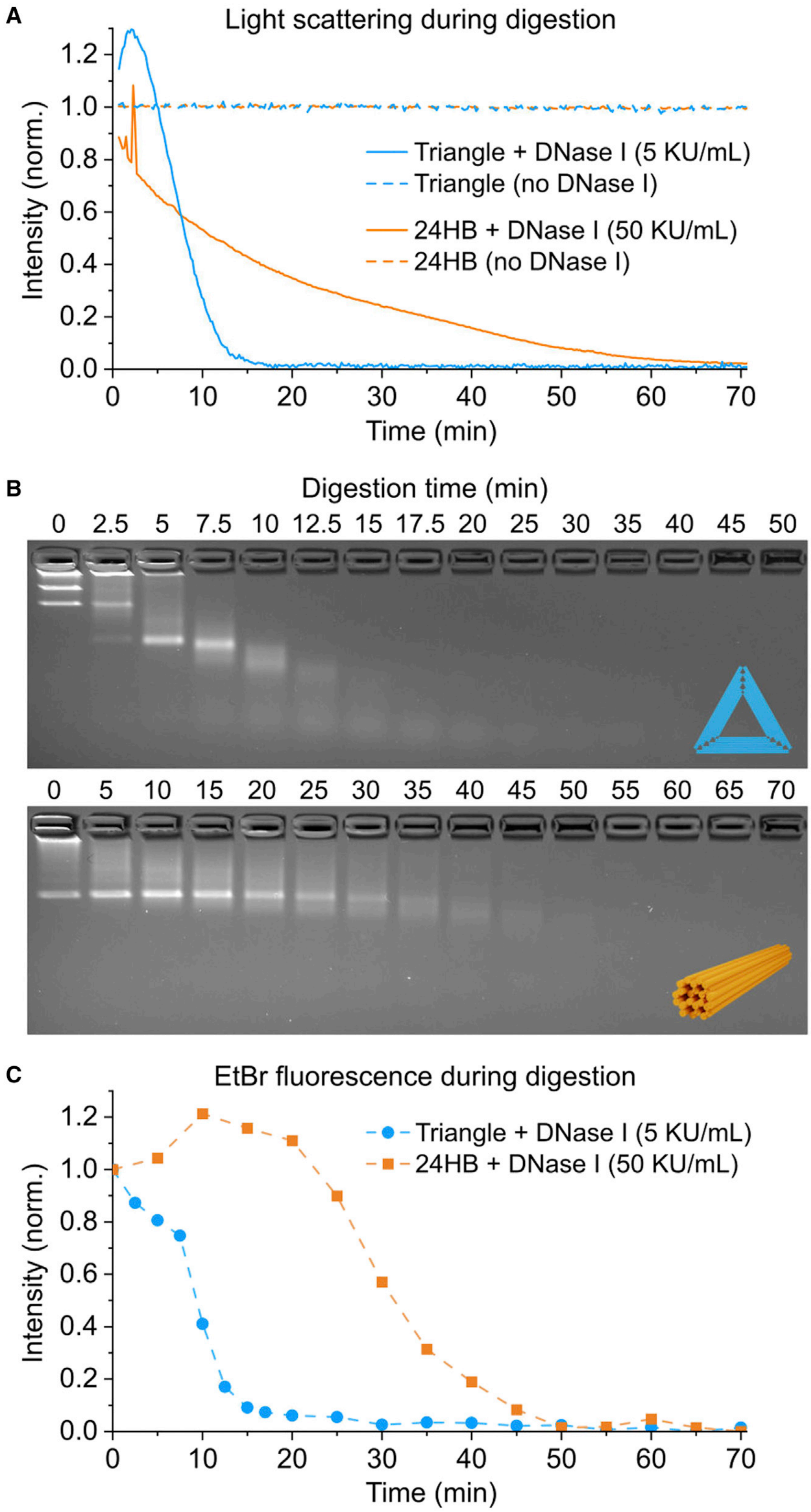

FIGURE 5 The DNase I digestion of the RTO and 24HB structures monitored by light scattering and AGE. (*A*) The light scattering intensities of 4 nM DNA origami structures during DNase I digestion, collected at 340 nm. Non-normalized digestion data are presented in Fig. S10. (*B*) An AGE analysis of digestion of the RTO (*top panel*) and 24HB (*bottom*) at 5 KU/mL DNase I concentration. The DNase I digestion is stopped at specified time points by an addition of SDS (the sample for 0 min digestion time contains no DNase I). (*C*) Analysis of the ethidium bromide fluorescence decay (calculated as total intensity per lane) in AGE during DNase I digestion. To see this figure in color, go online.

The digestion profiles obtained from the light-scattering measurements are compared with an AGE analysis carried out by quenching the digestion at set time points with SDS and analyzing the migration of the structures on an EtBr-stained agarose gel (Fig. 5, *B* and *C*). Here, the digestion causes both a dimming and the eventual disappearance

of the EtBr fluorescence and an increase of the electrophoretic mobility. The migration rate of the particles (Fig. 5 *B*) in the gel is related to their geometry and size, while the total EtBr fluorescence in each lane (Fig. 5 *C*) is proportional to the amount of intact DNA basepairs that are accessible for EtBr intercalation. The digestion profiles obtained from the AGE analysis are in line with the real-time monitoring by light scattering (Fig. 5 *A*). Subtle differences in the scattering and EtBr intensities, in particular during the initial phases of the digestion, reflect the differences between scattering as a direct method for studying the global decrease of the DNA origami size during digestion and the indirect detection with an intercalating reporter dye on AGE. Both this comparison and the comparison of scattering intensities versus qPCR measurements during folding (Figs. S4–S9) highlight the complementary nature of these methods as reporters of both global and local structural changes during assembly and disassembly—the scattering intensity depends on the molecular mass of the structure, while the intercalating dyes give information on the amount of hybridized strands that are not necessarily incorporated into the DNA origami structure.

CONCLUSION

We have presented a simple, label-free, and user-friendly method for monitoring DNA origami assembly and disassembly processes, i.e., folding/unfolding and enzymatic digestion of DNA origami. The technique is based on light scattering, and it can be performed on commonly accessible equipment like fluorometers or dynamic-light-scattering instruments. The procedure is straightforward, and it offers information on the temperatures at which the folding takes place and how fast it is. This information can be used to optimize DNA origami folding protocols for individual designs that result in faster processing times and higher yields. Although we show that optimized protocols help avoid aggregation during folding, we still recommend confirming the quality of the final product with the standard methods (AGE and TEM/AFM), as light scattering cannot directly differentiate between monomeric origami structures and higher-order multimers or aggregates. Multi-angle light-scattering measurements could offer more information on the shape of our samples or their state of aggregation. However, in that case, rigorous sample and measurement optimization is required to obtain data of sufficient quality, while imaging methods often provide the same or more information with less experimental and analysis effort.

In the field of DNA nanotechnology, advances in the development of methods often go hand in hand with increasing complexity of DNA nanostructure designs. Therefore, we believe that our approach will enable more researchers to implement folding procedure optimization as a standard part of their design process.

SUPPORTING MATERIAL

Supporting material can be found online at https://doi.org/10.1016/j.bpj.2022.10.036.

AUTHOR CONTRIBUTIONS

H.I. and G.P. carried out the experiments. All authors designed the research, analyzed the data, and wrote the article.

ACKNOWLEDGMENTS

We thank V. Aseyev, E. Riedle, and H. Derondeau for technical assistance and fruitful discussions. We thank A. Kucinic and C.E. Castro, who kindly provided us with staple mixtures and scaffolds for the four structures shown in Fig. 4. Financial support by the Emil Aaltonen Foundation, the Sigrid Jusélius Foundation, the Jane and Aatos Erkko Foundation, and ERC consolidator grant “DNA functional lattices” (project ID: 818635) is gratefully acknowledged. This work was carried out under the Academy of Finland Centers of Excellence Program (2022–2029) in Life-Inspired Hybrid Materials (LIBER), project number (346110). We acknowledge the provision of facilities and technical support by Aalto University Bioeconomy Facilities and OtaNano – Nanomicroscopy Center (Aalto-NMC) and Micronova Nanofabrication Center.

DECLARATION OF INTERESTS

The authors declare no competing interests.

REFERENCES

1. Seeman, N. C. 1982. Nucleic acid junctions and lattices. *J. Theor. Biol.* 99:237–247.
2. Seeman, N. C., and H. F. Sleiman. 2018. DNA nanotechnology. *Nat. Rev. Mater.* 3, 17068.
3. Chen, Y.-J., B. Groves, ..., G. Seelig. 2015. DNA nanotechnology from the test tube to the cell. *Nat. Nanotechnol.* 10:748–760.
4. Surana, S., A. R. Shenoy, and Y. Krishnan. 2015. Designing DNA nanodevices for compatibility with the immune system of higher organisms. *Nat. Nanotechnol.* 10:741–747.
5. Keller, A., and V. Linko. 2020. Challenges and perspectives of DNA nanostructures in biomedicine. *Angew. Chem. Int. Ed. Engl.* 59:15818–15833.
6. Heuer-Jungemann, A., and V. Linko. 2021. Engineering inorganic materials with DNA nanostructures. *ACS Cent. Sci.* 7:1969–1979.
7. Rothemund, P. W. K. 2006. Folding DNA to create nanoscale shapes and patterns. *Nature.* 440:297–302.
8. Andersen, E. S., M. Dong, ..., J. Kjems. 2009. Self-assembly of a nanoscale DNA box with a controllable lid. *Nature.* 459:73–76.
9. Douglas, S. M., H. Dietz, ..., W. M. Shih. 2009. Self-assembly of DNA into nanoscale three-dimensional shapes. *Nature.* 459:414–418.
10. Dietz, H., S. M. Douglas, and W. M. Shih. 2009. Folding DNA into twisted and curved nanoscale shapes. *Science.* 325:725–730.
11. Glaser, M., S. Deb, ..., D. M. Smith. 2021. The art of designing DNA nanostructures with CAD software. *Molecules.* 26:2287.
12. Dey, S., C. Fan, ..., P. Zhan. 2021. DNA origami. *Nat. Rev. Methods Primers.* 1:13.
13. Nummelin, S., B. Shen, ..., V. Linko. 2020. Robotic DNA nanostructures. *ACS Synth. Biol.* 9:1923–1940.

14. DeLuca, M., Z. Shi, …, G. Arya. 2020. Dynamic DNA nanotechnology: toward functional nanoscale devices. *Nanoscale Horiz.* 5:182–201.

15. Ijäs, H., I. Hakaste, …, V. Linko. 2019. Reconfigurable DNA origami nanocapsule for pH-controlled encapsulation and display of cargo. *ACS Nano.* 13:5959–5967.

16. Piskunen, P., R. Latham, …, V. Linko. 2022. Integrating CRISPR/Cas systems with programmable DNA nanostructures for delivery and beyond. *iScience.* 25, 104389.

17. Kuzyk, A., R. Schreiber, …, T. Liedl. 2012. DNA-based self-assembly of chiral plasmonic nanostructures with tailored optical response. *Nature.* 483:311–314.

18. Pilo-Pais, M., G. P. Acuna, …, T. Liedl. 2017. Sculpting light by arranging optical components with DNA nanostructures. *MRS Bull.* 42:936–942.

19. Shen, B., M. A. Kostiainen, and V. Linko. 2018. DNA origami nanophotonics and plasmonics at interfaces. *Langmuir.* 34:14911–14920.

20. Liu, N., and T. Liedl. 2018. DNA-assembled advanced plasmonic architectures. *Chem. Rev.* 118:3032–3053.

21. Zhang, T., C. Hartl, …, T. Liedl. 2018. 3D DNA origami crystals. *Adv. Mater.* 30, 1800273.

22. Wagenbauer, K. F., C. Sigl, and H. Dietz. 2017. Gigadalton-scale shape-programmable DNA assemblies. *Nature.* 552:78–83.

23. Wintersinger, C. M., D. Minev, …, W. M. Shih. 2022. Multi-micron crisscross structures from combinatorially assembled DNA-origami slats. Preprint at bioRxiv. https://doi.org/10.1101/2022.01.06.475243.

24. Xin, Y., B. Shen, …, A. Keller. 2021. Scaling up DNA origami lattice assembly. *Chemistry.* 27:8564–8571.

25. Sobey, T. L., S. Renner, and F. C. Simmel. 2009. Assembly and melting of DNA nanotubes from single-sequence tiles. *J. Phys. Condens. Matter.* 21, 034112.

26. Dunn, K. E., F. Dannenberg, …, J. Bath. 2015. Guiding the folding pathway of DNA origami. *Nature.* 525:82–86.

27. Rossi-Gendron, C., F. El Fakih, …, D. Baigl. 2022. Isothermal self-assembly of multicomponent and evolutive DNA nanostructures. Preprint at chemRxiv. https://doi.org/10.26434/chemrxiv–2022–12jqs.

28. Sobczak, J.-P. J., T. G. Martin, …, H. Dietz. 2012. Rapid folding of DNA into nanoscale shapes at constant temperature. *Science.* 338:1458–1461.

29. Bae, W., K. Kim, …, T.-Y. Yoon. 2014. Programmed folding of DNA origami structures through single-molecule force control. *Nat. Commun.* 5:5654.

30. Wah, J. L. T., C. David, …, A. Estevez-Torres. 2016. Observing and controlling the folding pathway of DNA origami at the nanoscale. *ACS Nano.* 10:1978–1987.

31. Huang, P., J. Wang, …, H. Pei. 2019. A "time-frozen" technique in microchannel used for the thermodynamic studies of DNA origami. *Biosens. Bioelectron.* 131:224–231.

32. Fischer, S., C. Hartl, …, B. Nickel. 2016. Shape and interhelical spacing of DNA origami nanostructures studied by small-angle X-ray scattering. *Nano Lett.* 16:4282–4287.

33. Wei, X., J. Nangreave, …, Y. Liu. 2013. Mapping the thermal behavior of DNA origami nanostructures. *J. Am. Chem. Soc.* 135:6165–6176.

34. Schneider, F., N. Möritz, and H. Dietz. 2019. The sequence of events during folding of a DNA origami. *Sci. Adv.* 5, eaaw1412.

35. Majikes, J. M., P. N. Patrone, …, J. A. Liddle. 2020. Revealing thermodynamics of DNA origami folding via affine transformations. *Nucleic Acids Res.* 48:5268–5280.

36. Ijäs, H., B. Shen, …, V. Linko. 2021. Unraveling the interaction between doxorubicin and DNA origami nanostructures for customizable chemotherapeutic drug release. *Nucleic Acids Res.* 49:3048–3062.

37. Stahl, E., T. G. Martin, …, H. Dietz. 2014. Facile and scalable preparation of pure and dense DNA origami solutions. *Angew. Chem. Int. Ed. Engl.* 53:12735–12740.

38. Huang, C.-M., A. Kucinic, …, C. E. Castro. 2021. Integrated computer-aided engineering and design for DNA assemblies. *Nat. Mater.* 20:1264–1271.

Biophysical Journal, Volume *121*

Supplemental information

A label-free light-scattering method to resolve assembly and disassembly of DNA nanostructures

Heini Ijäs, Tim Liedl, Veikko Linko, and Gregor Posnjak

Supplementary Information for
A label-free light scattering method to resolve assembly and disassembly of DNA nanostructures

Heini Ijäs[1,2], **Tim Liedl**[2], **Veikko Linko**[1,3,*] **& Gregor Posnjak**[2,*]

[1]Biohybrid Materials, Department of Bioproducts and Biosystems, Aalto University, P.O. Box 16100, 00076 Aalto, Finland
[2]Faculty of Physics and Center for NanoScience (CeNS), Ludwig-Maximilians-University, Geschwister-Scholl-Platz 1, 80539 Munich, Germany
[3]LIBER Center of Excellence, Aalto University, P.O. Box 15100, 00076 Aalto, Finland

Contents

Page

Supplementary notes **3**

Note S1: Spectrum of detected light
(Supplementary Figure S1) 3

Note S2: Scattering intensities at different wavelengths during folding
(Supplementary Figure S2) 4

Note S3: Background signals from buffer, staples and scaffold
(Supplementary Figure S3) 5

Note S4: qPCR vs. light scattering
(Supplementary Figures S4-S9) 6

Note S5: Non-normalized scattering intensities during DNase I digestion
(Supplementary Figure S10) 12

Supplementary notes

Note S1: Spectrum of detected light.

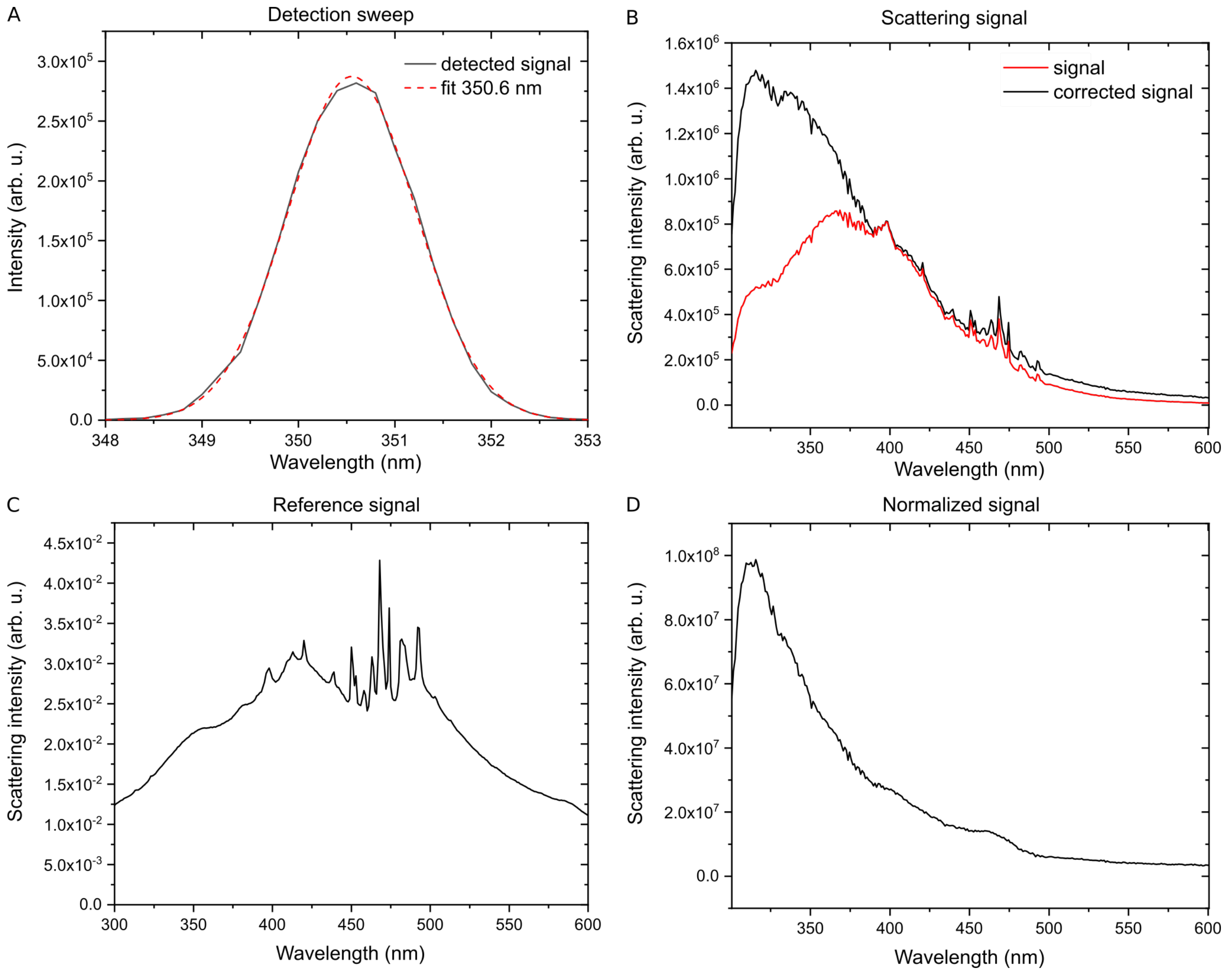

Supplementary Figure S 1. Detected scattered light intensity for different measurement configurations. (**A**) Offset of the detection path monochromator. This can be measured by setting a fixed excitation wavelength (here 350 nm) and doing a narrow sweep of the detection monochromator. The position of the measured scattering peak can then be used as the offset of the detection monochromator. (**B**) Wavelength dependence of the scattering intensity can be measured with a synchronous sweep of both the excitation and detection monochromators. The red curve shows the detected scattering signal at different wavelengths of excitation and detection. The black curve is the same signal, but corrected by a wavelength dependent factor that accounts for the wavelength-dependent sensitivity of the detector. We can see that the correction is most striking for wavelengths below 400 nm, as the photomultiplier used in our system is less sensitive in that range. The sharp peaks in the range 400–500 nm are caused by spectral lines of the light source. (**C**) Graph of the reference signal, *i.e.* the lamp intensity at different wavelengths. We can clearly see the spectral lines of the light source which caused additional peaks in (B). We can also see that the intensity of the lamp is diminished below 400 nm. (**D**) Corrected scattering signal normalized by the reference signal. We can see that after applying the wavelength-dependent corrections the scattering intensity is increasing with decreasing wavelengths. However, the strong scattering signal at short wavelengths is a consequence of the numerical corrections, so for better signal to noise ratio it is better to do the measurement at wavelengths where both the raw scattering signal in (B) and the lamp intensity in (C) are relatively high.

Note S2: Scattering intensities at different wavelengths during folding.

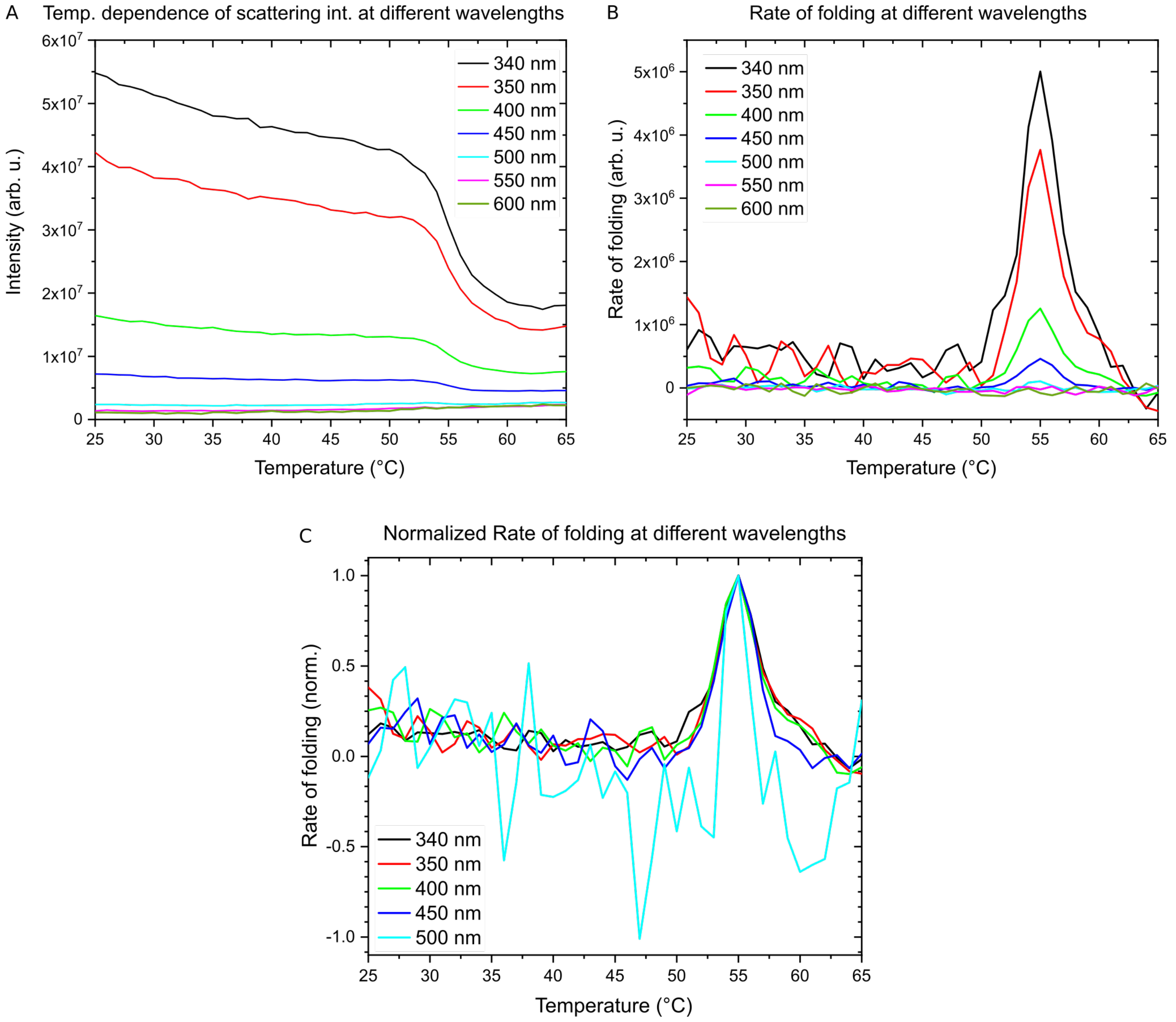

Supplementary Figure S 2. Folding of the tetrapod structure with a cooling rate of 20 min/℃. (**A**) Scattering intensity values at different wavelengths. (**B**) Temperature derivatives of the scattering intensities show that with increasing wavelength the scattering peak in the Rate-of-folding curves is becoming less and less pronounced. (**C**) The normalized Rate-of-folding curves (from (B)) show that the scattering peaks have a very similar shape for peaks at different wavelengths, but the signal-to-noise ration decreases, which can be compensated for by longer exposure times. Curves for wavelengths above 500 nm are omitted because of their poor signal-to-noise ratio.

Note S3: Background signals from buffer, staples and scaffold.

When monitoring folding and unfolding processes with techniques like qPCR and UV absorption measurements, the scaffold and the staples can produce considerable background signal. To check for this, we prepared different mixtures omitting one or more of the folding components and ran repeated heating and cooling cycles. Fig. S3A shows repeated cycles of heating and cooling for a cuvette filled only with a typical folding buffer. In Fig. S3B a similar measurement was done for a mixture of all the staples of the tetrapod structure in the same buffer and for Fig. S3C we used only the scaffold in buffer, at the same concentration as in a folding mixture. Out of the three measurements the highest signal comes as expected from the scaffold, as it has the highest molecular weight of around 2.5 MDa. None of the three samples show behaviour which would resemble the increase in intensity during folding of DNA origami (*i.e.* an increase in intensity during cooling of the sample typically in the 50 °C to 60 °C range and a decrease in intensity at a somewhat higher temperature while heating the sample), but they do exhibit an increase in intensity upon heating the sample above 60 °C. We do not have a conclusive answer to the cause of this increase, but we hypothesise it is at least partially due to formation of gas bubbles in the liquid because of the reduced solubility of gasses at high temperatures. This is supported by the observation that the high scattering intensity decreases over time if incubating at high temperature, probably because gas bubbles start to leave the liquid. We can see something similar when cycling the sample through several cycles of heating and cooling, where the maximum intensity at the highest temperature typically decreases with each cycle. It is possible that the scaffold somehow contributes to this high temperature increase as we have noticed that this effect tends to be stronger in samples with the scaffold.

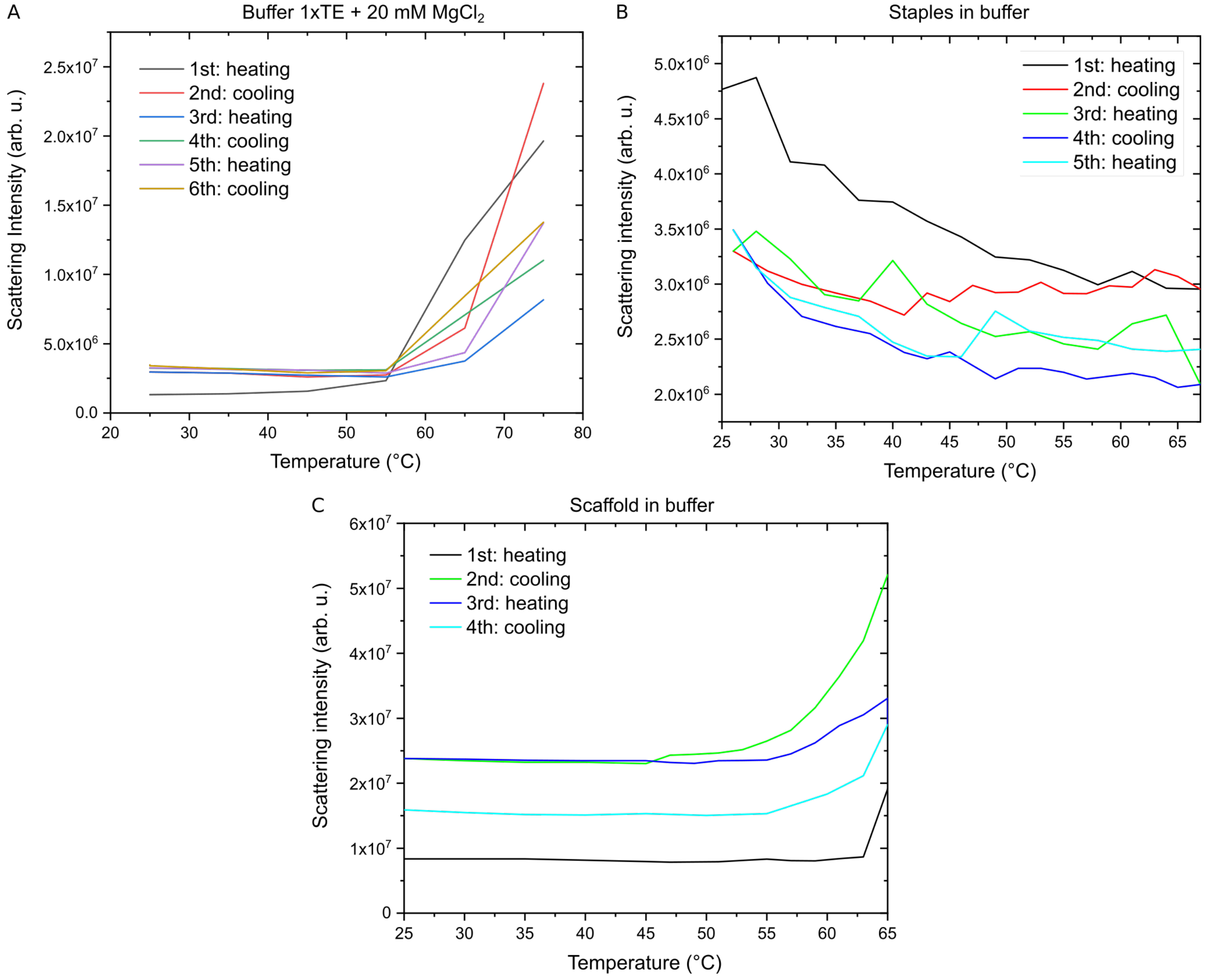

Supplementary Figure S 3. Background signals in a folding mixture. (**A**) Scattering intensity during 6 rounds of heating and cooling of a typical DNA origami folding buffer. (**B**) Scattering intensity during 5 rounds of heating and cooling of the tetrapod staples in a typical DNA origami folding buffer. (**C**) Scattering intensity during 4 rounds of heating and cooling a typical DNA origami scaffold at 10 nM in a typical DNA origami folding buffer.

Note S4: qPCR *vs.* light scattering.

For the qPCR measurements we prepared 100 μL of a folding mixture at 10 nM scaffold and 100 nM staple concentration in 1×TE buffer with appropriate $MgCl_2$ concentration. Additionally we prepared 50 μL of two "blank" mixtures for each structure, where we would substitute either the scaffold or the staples with MilliQ water. All samples were stained with SyberSafe fluorescent intercalating dye at 4× concentration. We transferred the samples into an optical quality PCR tube strip in 25 μL aliqotes and ran three rounds of annealing and heating ramps on a Bio-Rad C1000 Touch with a CFX96 optical unit measuring the fluorescence at the end of each step. The measurements were then averaged between the different aliquotes and runs (for the folding mixture that means an average of 12 runs per structure) and the staple signal (which causes the most background) was subtracted from the full folding mixture. The rates of folding were obtained by taking the derivative of the averaged and subtracted measurements.

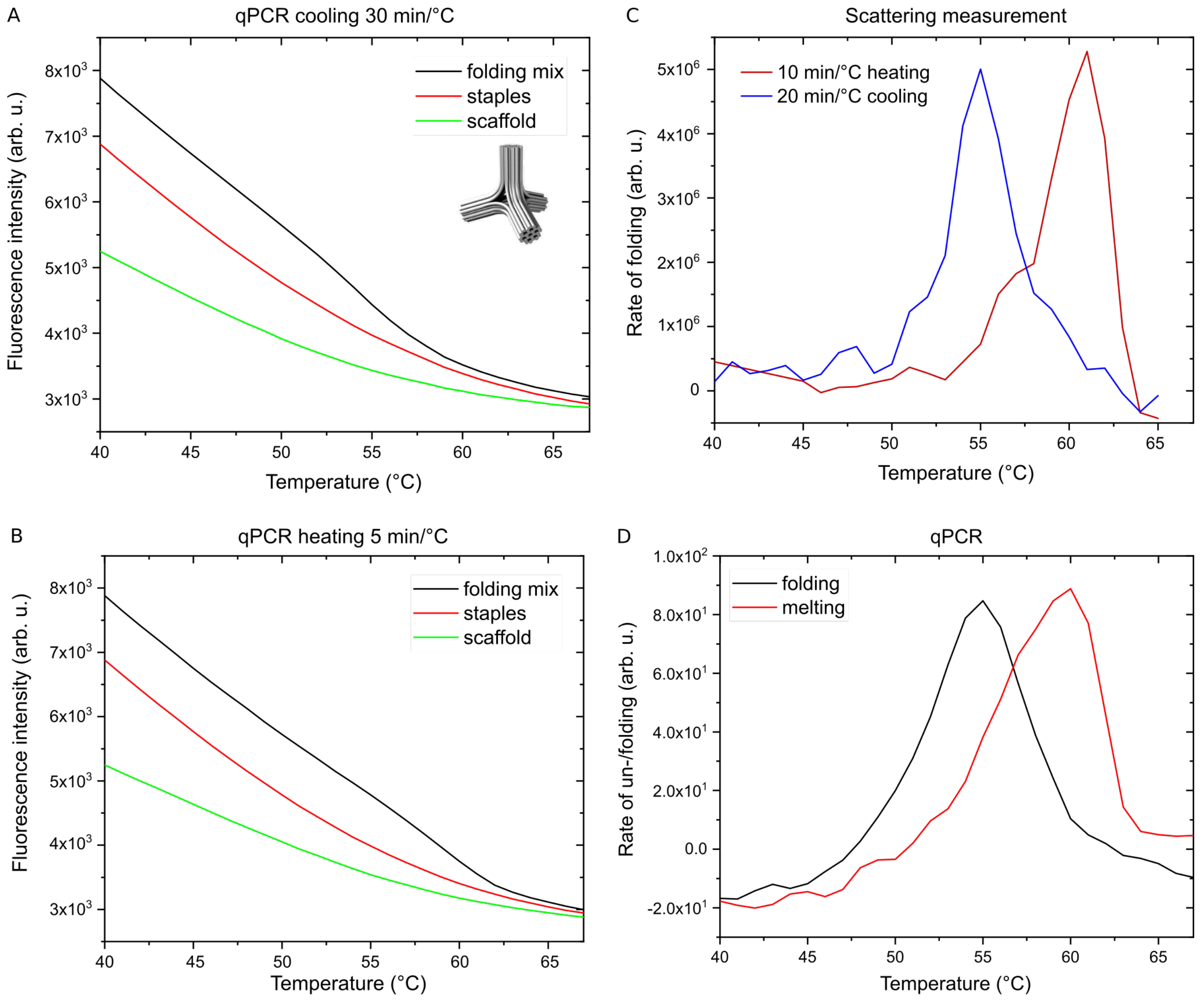

Supplementary Figure S 4. Folding of the "tetrapod" structure. (**A**) qPCR fluorescence intensity during cooling. (**B**) qPCR fluorescence intensity during heating. (**C**) Rate-of-folding and unfolding derived from scattering measurements. (**D**) Rate-of-folding and unfolding derived from qPCR measurements.

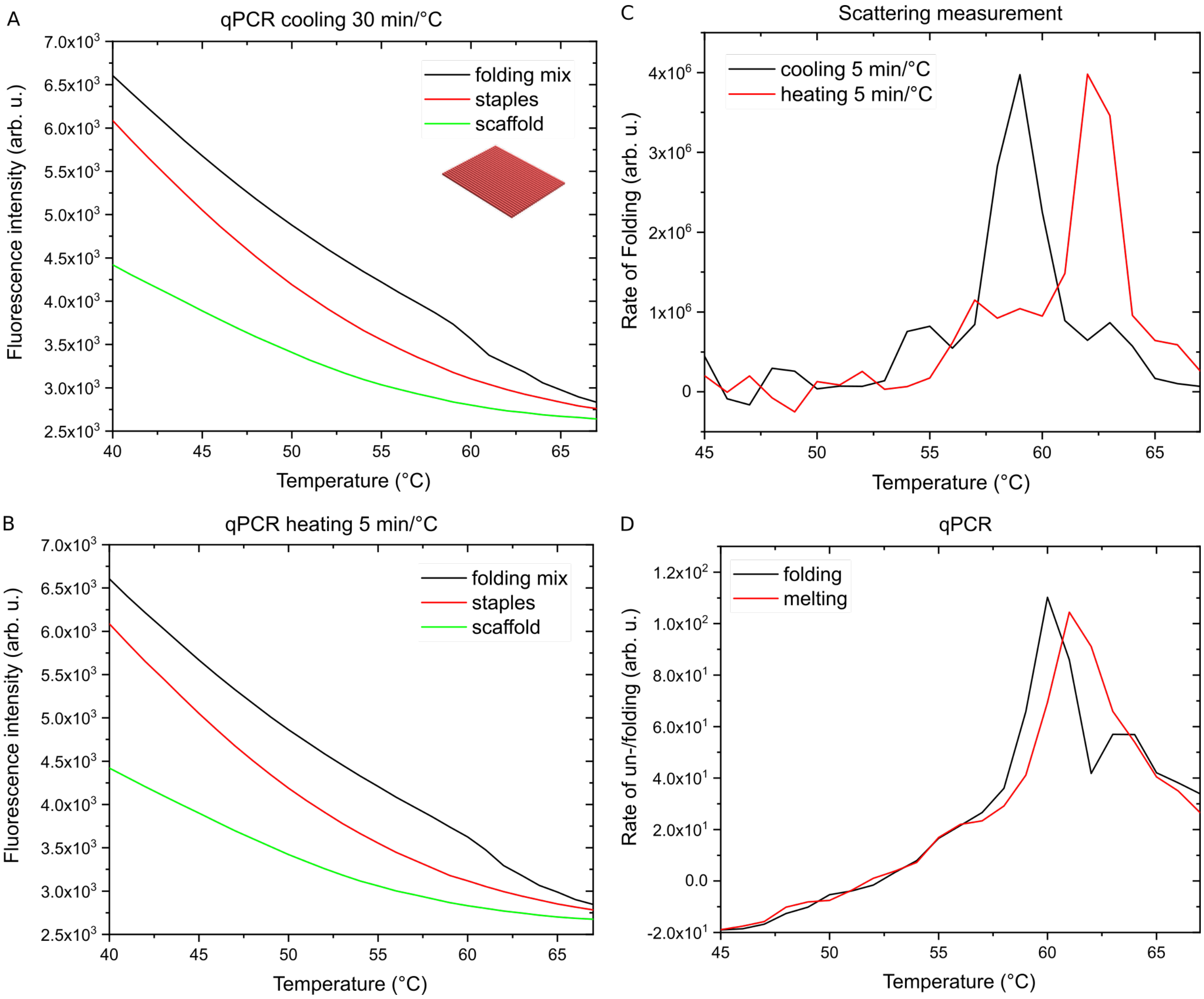

Supplementary Figure S 5. Folding of the RRO structure. (**A**) qPCR fluorescence intensity during cooling. (**B**) qPCR fluorescence intensity during heating. (**C**) Rate-of-folding and unfolding derived from scattering measurements. (**D**) Rate-of-folding and unfolding derived from qPCR measurements.

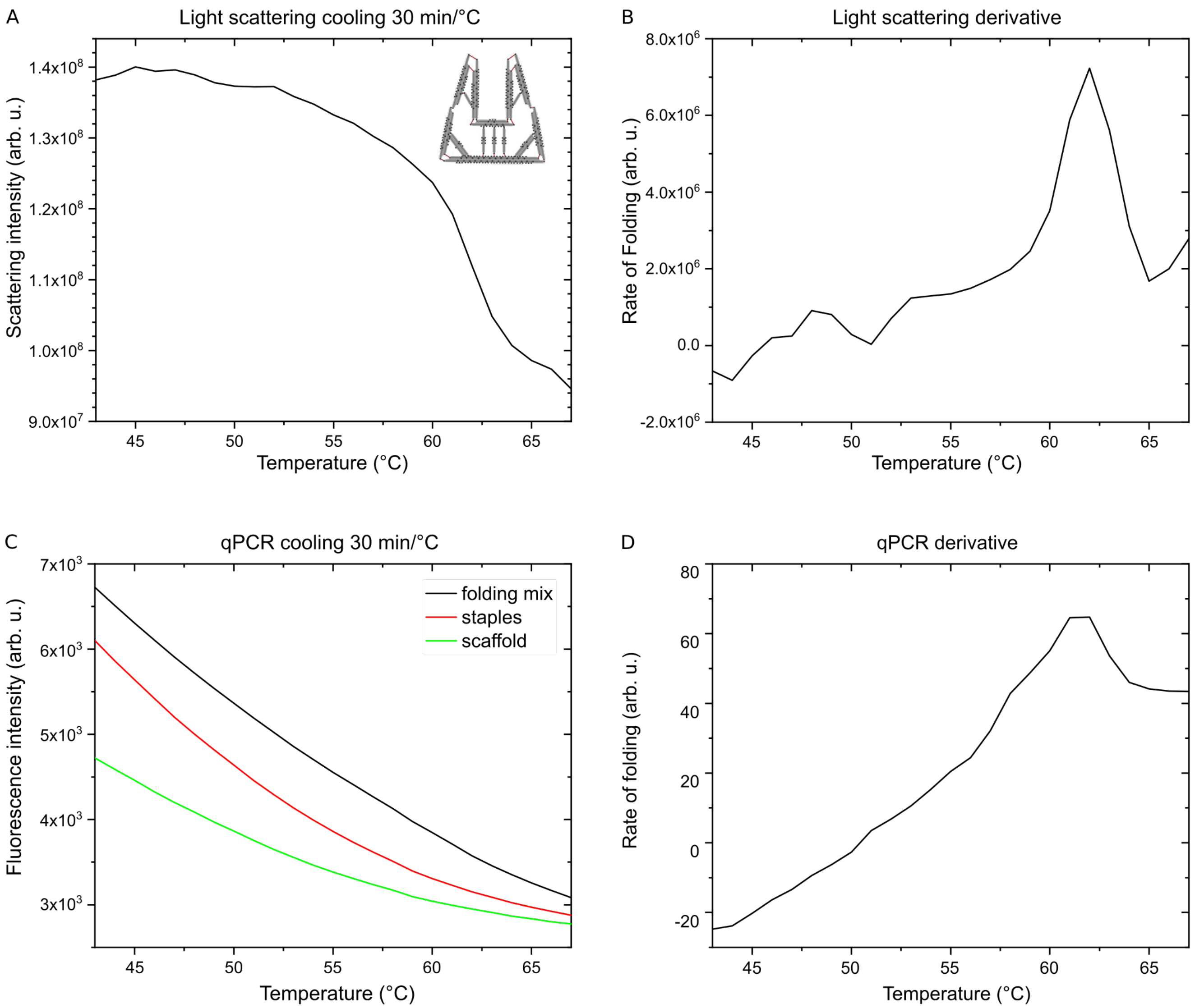

Supplementary Figure S 6. Folding of the "gripper" structure. (**A**) Scattering intensity during cooling. (**B**) Rate-of-folding derived from the scattering measurement. (**C**) qPCR fluorescence intensity during cooling. (**D**) Rate-of-folding derived from the qPCR measurements.

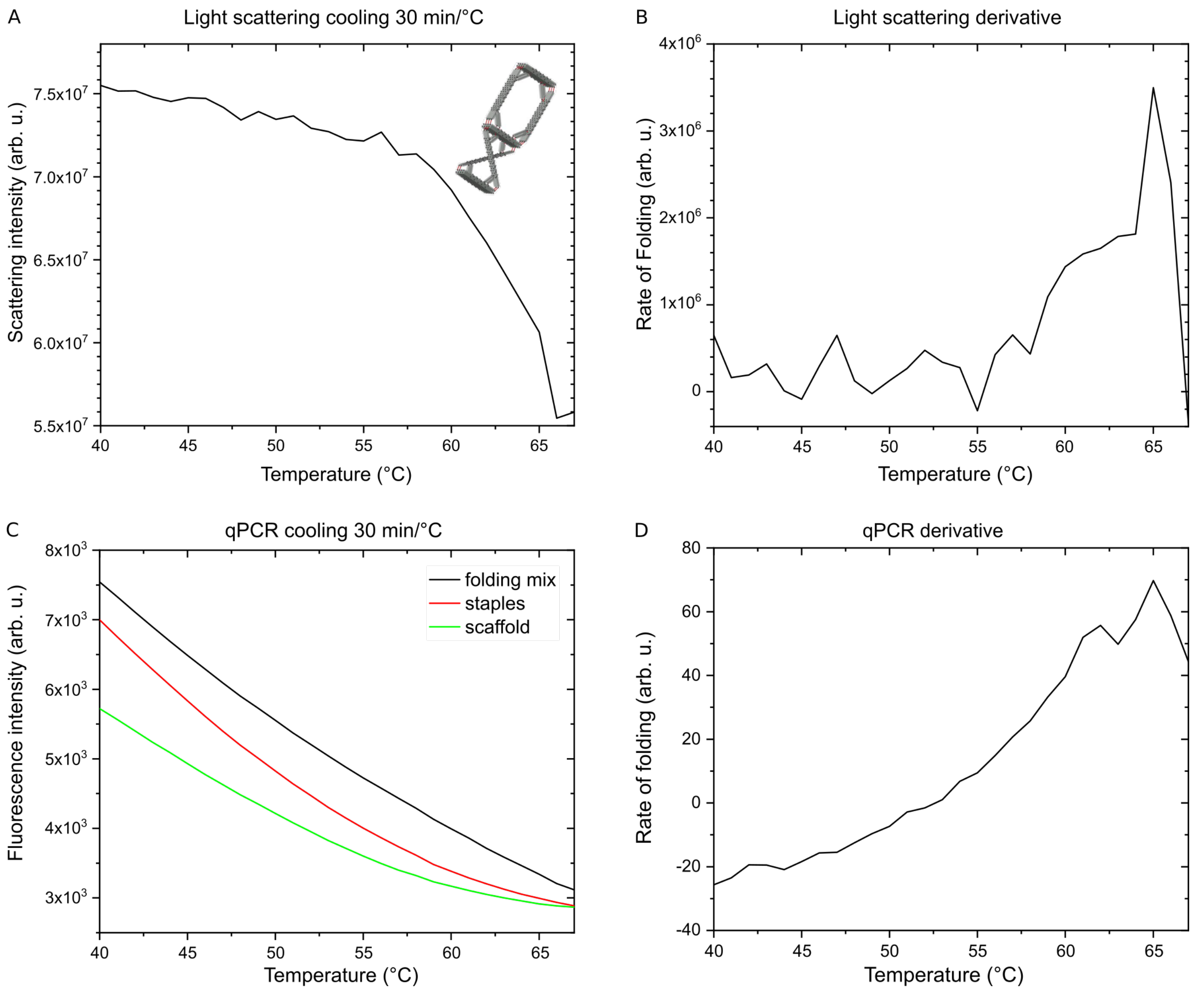

Supplementary Figure S 7. Folding of the "compliant compound joint" structure. (**A**) Scattering intensity during cooling. (**B**) Rate-of-folding derived from the scattering measurement. (**C**) qPCR fluorescence intensity during cooling. (**D**) Rate-of-folding derived from the qPCR measurements.

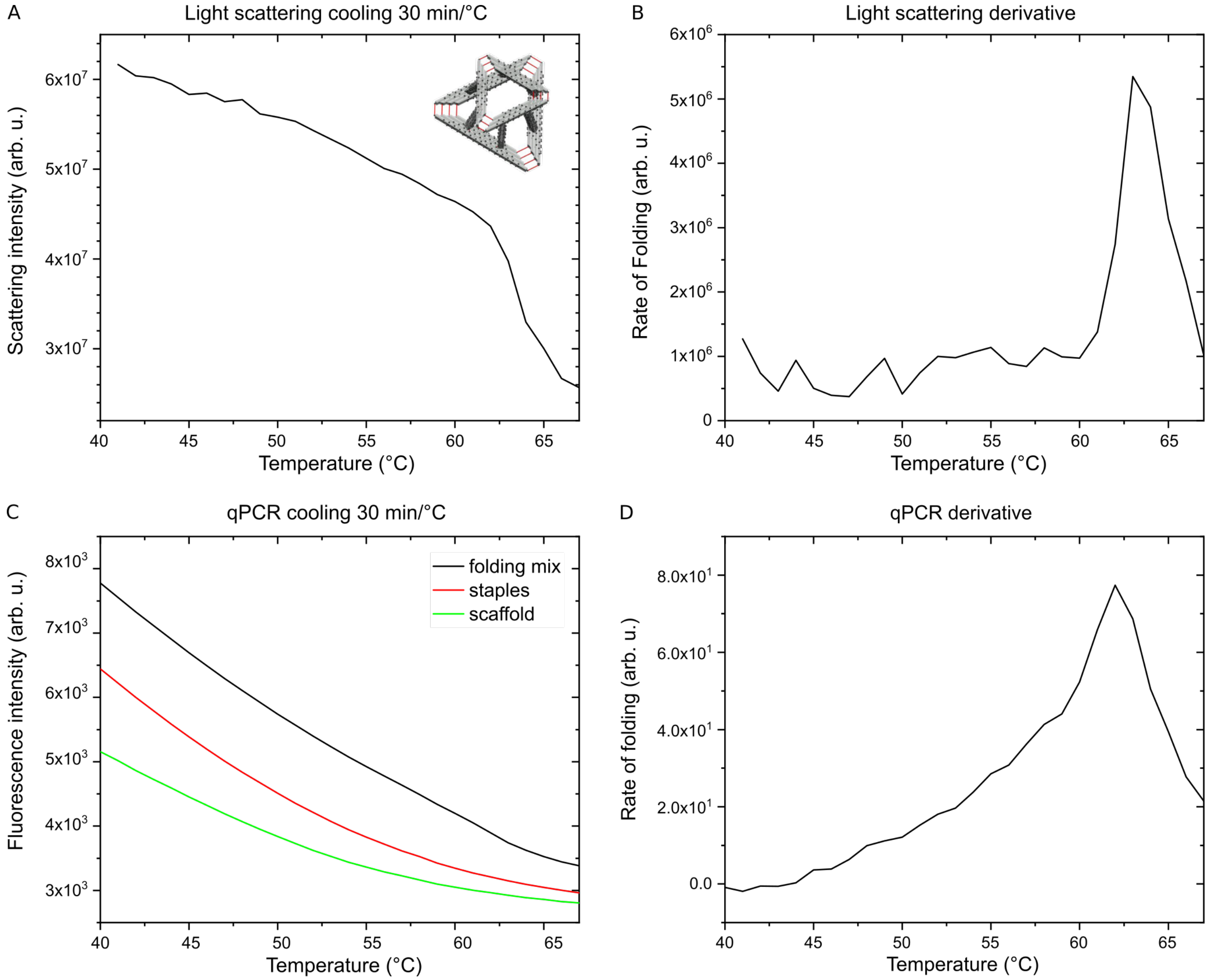

Supplementary Figure S 8. Folding of the "Stewart platform" structure. (**A**) Scattering intensity during cooling. (**B**) Rate-of-folding derived from the scattering measurement. (**C**) qPCR fluorescence intensity during cooling. (**D**) Rate-of-folding derived from the qPCR measurements.

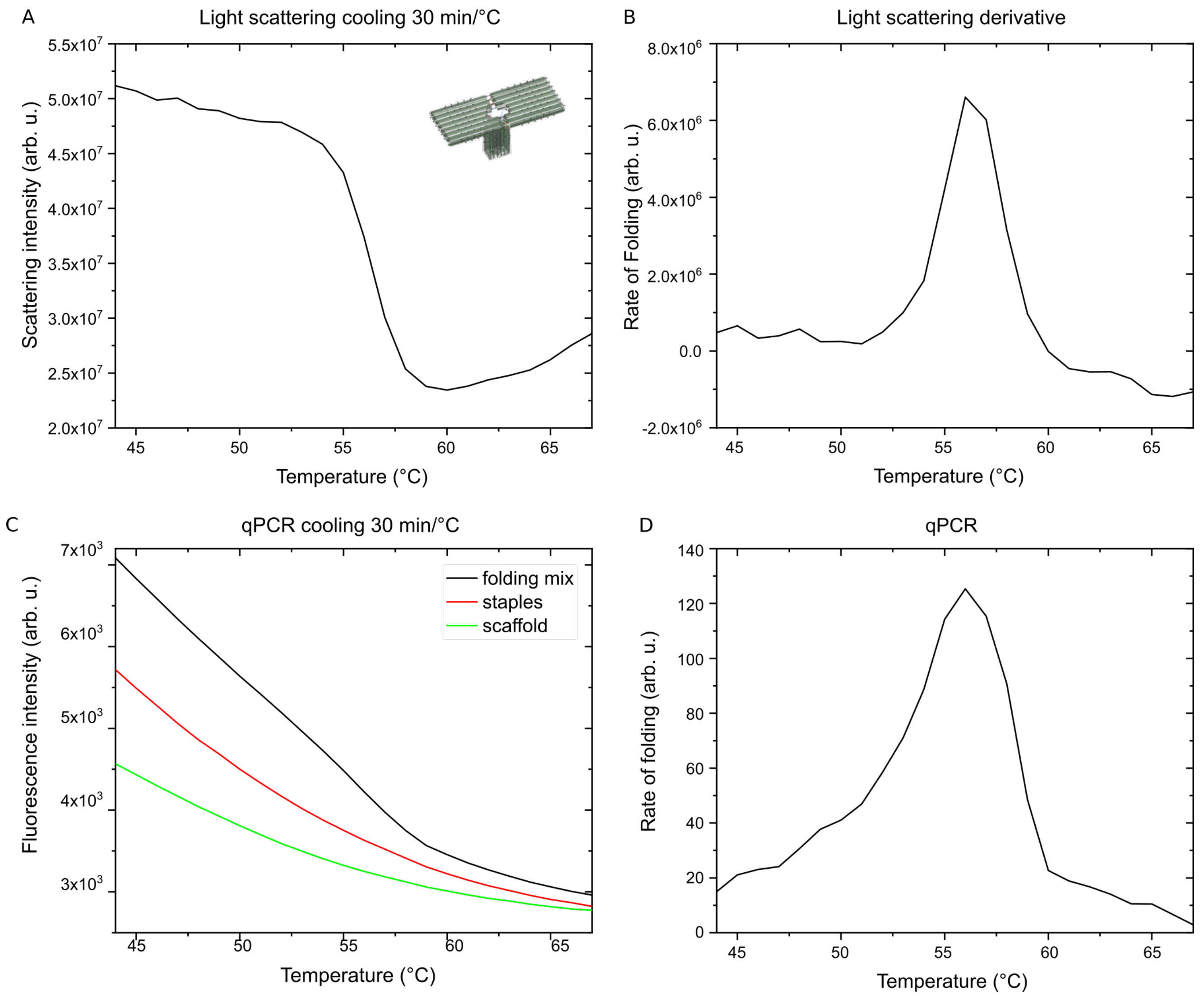

Supplementary Figure S 9. Folding of the "nanopore" structure. (**A**) Scattering intensity during cooling. (**B**) Rate-of-folding derived from the scattering measurement. (**C**) qPCR fluorescence intensity during cooling. (**D**) Rate-of-folding derived from the qPCR measurements.

Note S5: Non-normalized scattering intensities during DNase I digestion.

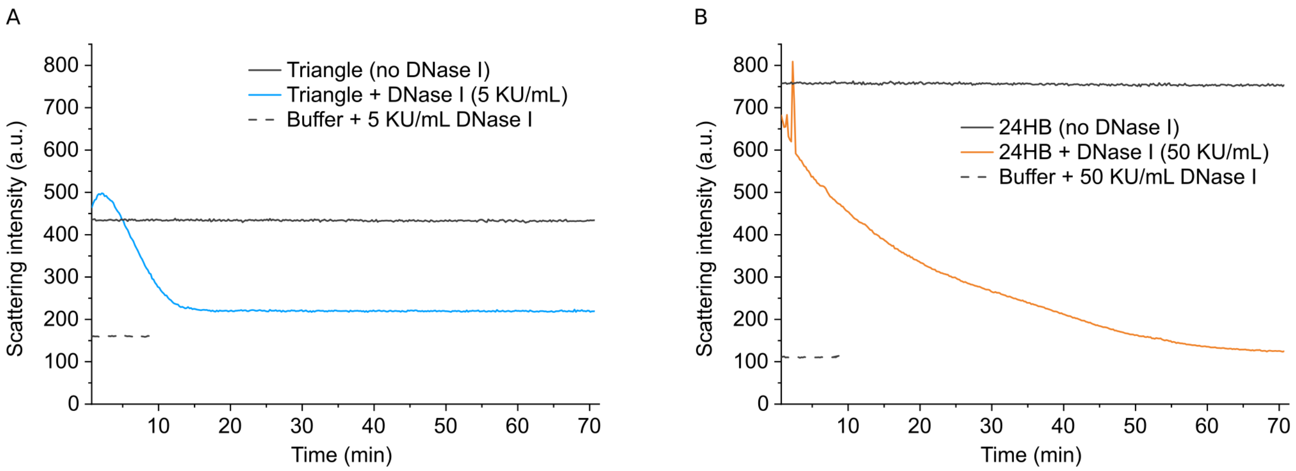

Supplementary Figure S 10. Scattering intensity values at 340 nm for RTO and 24HB origami structures, as presented in the Figure 5 in the main article, but without background subtraction and intensity normalization. The intensity values in panel A have been obtained with a PMT voltage of 650 V, while a 620 V voltage was used for the experiment in panel B to avoid saturating the detector when measuring the more strongly scattering 24HB. The intensity values thus cannot be directly compared between panels A and B. (**A**) The digestion of 4 nmol/L RTO in the presence of 5 KU/mL DNase I. The digestion curve is compared both to a sample with 4 nmol/L RTO without DNase I, and to a reference sample containing the digestion buffer and 5 KU/mL DNase I. (**B**) The digestion of 4 nmol/l 24HB with similar reference data sets as for the RTO.
